# ErbB2/HER2 receptor tyrosine kinase regulates human papillomavirus promoter activity

**DOI:** 10.3389/fimmu.2024.1335302

**Published:** 2024-02-02

**Authors:** Snježana Mikuličić, Merha Shamun, Annika Massenberg, Anna-Lena Franke, Kirsten Freitag, Tatjana Döring, Johannes Strunk, Stefan Tenzer, Thorsten Lang, Luise Florin

**Affiliations:** ^1^ Institute for Virology, University Medical Center of the Johannes Gutenberg-University Mainz, Mainz, Germany; ^2^ University of Bonn, Faculty of Mathematics and Natural Sciences, Life & Medical Sciences (LIMES) Institute, Bonn, Rheinland-Pfalz, Germany; ^3^ Institute for Immunology, University Medical Center of the Johannes Gutenberg-University Mainz, Mainz, Rheinland-Pfalz, Germany; ^4^ Helmholtz Institute for Translational Oncology (HI-TRON) Mainz, Mainz, Rheinland-Pfalz, Germany

**Keywords:** human papillomavirus, HPV16, promoter activity, ErbB2, HER2/neu, tyrosine kinase inhibitor, tucatinib, E6 E7 oncogene expression

## Abstract

Human papillomaviruses (HPVs) are a major cause of cancer. While surgical intervention remains effective for a majority of HPV-caused cancers, the urgent need for medical treatments targeting HPV-infected cells persists. The pivotal early genes E6 and E7, which are under the control of the viral genome’s long control region (LCR), play a crucial role in infection and HPV-induced oncogenesis, as well as immune evasion. In this study, proteomic analysis of endosomes uncovered the co-internalization of ErbB2 receptor tyrosine kinase, also called HER2/neu, with HPV16 particles from the plasma membrane. Although ErbB2 overexpression has been associated with cervical cancer, its influence on HPV infection stages was previously unknown. Therefore, we investigated the role of ErbB2 in HPV infection, focusing on HPV16. Through siRNA-mediated knockdown and pharmacological inhibition studies, we found that HPV16 entry is independent of ErbB2. Instead, our signal transduction and promoter assays unveiled a concentration- and activation-dependent regulatory role of ErbB2 on the HPV16 LCR by supporting viral promoter activity. We also found that ErbB2’s nuclear localization signal was not essential for LCR activity, but rather the cellular ErbB2 protein level and activation status that were inhibited by tucatinib and CP-724714. These ErbB2-specific tyrosine kinase inhibitors as well as ErbB2 depletion significantly influenced the downstream Akt and ERK signaling pathways and LCR activity. Experiments encompassing low-risk HPV11 and high-risk HPV18 LCRs uncovered, beyond HPV16, the importance of ErbB2 in the general regulation of the HPV early promoter. Expanding our investigation to directly assess the impact of ErbB2 on viral gene expression, quantitative analysis of E6 and E7 transcript levels in HPV16 and HPV18 transformed cell lines unveiled a noteworthy decrease in oncogene expression following ErbB2 depletion, concomitant with the downregulation of Akt and ERK signaling pathways. In light of these findings, we propose that ErbB2 holds promise as potential target for treating HPV infections and HPV-associated malignancies by silencing viral gene expression.

## 1 Introduction

Human Papillomaviruses (HPVs) are a group of small, nonenveloped viruses that preferentially infect epithelial cells in the skin and mucosa. While low-risk HPV types are known to cause benign warts such as condyloma, the oncogenic high-risk HPV types pose a significant health risk, contributing to the development of various cancers, including cervical, penile, anal as well as head and neck tumors. Notable examples of these high-risk HPV types are HPV16 and 18, which are strongly associated with malignant changes in keratinocytes (1–3).

The structure of papillomaviruses features a ~55 nm spherical capsid comprising as building blocks major capsid protein L1 and minor capsid protein L2 (4, 5). Inside resides the HPV genome, an 8 kb circular double-stranded DNA, containing a long control region (LCR) responsible for regulating viral replication and early gene transcription. The LCR contains essential elements such as the early promoter that governs the expression of the early expressed genes, including E6 and E7, critical for HPV’s oncogenic potential and suppression of the host immune response (6–12).

To successfully infect and replicate, HPVs require mitotically active epithelial cells. The entry of HPVs into cells occurs through clathrin-independent endocytosis, which involves multiple signaling events leading to membrane protein clustering, cytoplasmic endocytic factor recruitment, and actin remodeling (13–20). The entry receptor complex for HPV includes various molecules such as tetraspanin CD151, laminin-binding integrins, growth-factor receptors, and the annexin A2 heterotetramer (21–26). Following virus internalization, the HPV capsid undergoes disassembly, enabling L2 membrane translocation and further transport of the infectious complex towards the nucleus. After mitosis, this complex enters the nucleus and locates within promyelocytic leukemia (PML) nuclear bodies, where viral gene transcription and replication takes place (19, 27, 28). Despite significant advancements in understanding HPV infection, many molecules involved in the HPV entry and early gene regulation remain unidentified.

A protein family of high significance in pathogen infection is the erythroblastic leukemia viral oncogene homologue (ErbB) growth factor receptor family consisting of the four members ErbB1, ErbB2, ErbB3, and ErbB4 (29). ErbB1, also known as the epidermal growth factor receptor (EGFR), is the most studied family member. It promotes the infection of a large number of viruses, including influenza A virus, hepatitis B/C virus, coronaviruses, herpesviruses, and papillomaviruses. Manipulating EGFR activation by down-regulation might provide implications for therapeutic interventions (29–33). Following ligand binding, ErbB signaling is mediated through oligomerization and phosphorylation of intracellular domains, resulting in the activation of major intracellular signaling pathways, including the phosphatidylinositol 3-kinase (PI3K)/Akt and the mitogen-activated protein kinase (MAPK)/extracellular signal-regulated kinase 1/2 (ERK1/2, collectively termed ERK) pathways (34, 35), in the following briefly called Akt and ERK. Both the Akt and the ERK pathway have been shown to play crucial roles in HPV16 infection steps including entry platform formation, endocytic vesicle maturation, and viral gene expression (14, 16, 18, 20, 36–38). In other studies, it has been shown that the full-length, or a cleaved part of the ErbB-molecule, might translocate into the nucleus via its nuclear localization signal (NLS) and bind directly to DNA for transcriptional regulation (39–44).

ErbB2, alternatively referred to as HER2 or neu, interacts with EGFR on the cell surface and plays an important role in both Akt and ERK signaling pathways (45–49). ErbB2 typically does not spontaneously form homo-oligomers, but overexpression can result in the functional formation of homo-dimers (34). Upregulation of ErbB2 expression level or activity are known oncogenic drivers in various human malignancies (50). Two tyrosine kinase inhibitors (TKI), tucatinib (sold under the brand name Tukysa) and CP-724714, specifically inhibit ErbB2 activation and its downstream signaling pathways. Such TKIs prevent phosphorylation and activation of signal transduction by competing for the ATP-binding domain of protein kinases (49, 51, 52). Both inhibitors show high selectivity for ErbB2 with minimal inhibition of EGFR activity. While CP-724714 shows side effects and low efficiencies (https://classic.clinicaltrials.gov/ct2/show/NCT00102895), tucatinib is approved by the United States Food and Drug Administration (FDA) and widely used for the treatment of metastatic ErbB2/HER2-positive solid cancers (47, 53). To the best of our knowledge, the involvement of ErbB2/HER2 and its targeting by tucatinib or CP-724714 has never been studied in the context of HPV infection, neither in entry nor in early promoter activity.

In this study, we identified a proteome network related to internalized HPV16 pseudoviruses (PsVs) into keratinocytes that exhibits, among other cellular factors, ErbB2 as central component. Driven by this insight, we aimed for elucidating at which HPV infection steps ErbB2 is involved. By modulating ErbB2 expression levels and pharmacologically inhibiting its activation, we investigated the impact of ErbB2 on HPV16 binding, entry platform formation, viral genome delivery to PML nuclear bodies, ErbB2-mediated Akt and ERK signaling, and LCR promoter activity of not exclusively HPV16, but as well HPV18 and HPV11. Furthermore, we expanded our investigations to include HPV16 and HPV18 infected and transformed cell lines, namely CaSki and HeLa, to assess the impact of ErbB2 on viral gene expression.

## 2 Materials and methods

### 2.1 Antibodies, inhibitors, and plasmids

HPV16 L1 mouse monoclonal antibody (mAb) 16L1-312F and rabbit polyclonal antibody (pAb) K75 have been previously described (54–56). Rab5-specific mouse mAb (sc-46692; D-11) and ErbB2/HER2-specific mouse mAb recognizing N-terminus (sc-08; 9G6) for CLSM experiments were purchased from Santa Cruz Biotechnology (Heidelberg, Germany). A rabbit pAb raised against CD151 (ab185684) used for STED experiments was purchased from Abcam (Amsterdam, Netherlands) and a rabbit anti-CD151 serum generated against the recombinant large extracellular loop of CD151 (rCD151) used for WB after non-reducing SDS-PAGE was a kind gift from Fedor Berditchevski (University of Birmingham, United Kingdom). β-actin (A5441) and α-tubulin (B-5-1-2) specific mouse mAbs were from Sigma-Aldrich (St. Louis, MO, USA). Rabbit mAbs specific for ErbB2 (clone 29D8), p-ErbB2 (Y877), p-ErbB2 (Y1221/1222) clone 6B12, total ERK1/2 (p44/42) MAPK; clone 137F5), p-ERK1/2 (T202/Y204), clone D13.14.4E, total Akt, clone C67E7, p-Akt (S473), clone D9E, EGFR clone D38B1, and p-EGFR (Y1068), clone D7A5, were purchased from Cell Signaling (Leiden, Netherlands). Rabbit mAb PML (sc-5621) was purchased from Santa Cruz Biotechnology. Horseradish peroxidase-coupled (HRP) secondary antibodies for immunoblot were from Dianova (Hamburg, Germany). Secondary antibodies for STED microscope were goat anti-rabbit coupled to STARRED (STRED-1002, Abberior Instruments, Goettingen, Germany) and donkey anti-mouse coupled to Alexa594 (A-21203) and other Alexa-conjugated secondary antibodies were provided from Invitrogen (Carlsbad, CA, USA).

CP-724714 was obtained from Selleckchem (Houston, TX, USA) and tucatinib from MedKoo Biosciences (Morrisville, NC, USA). Control cells were treated with inhibitor dissolvent, dimethyl sulfoxide (DMSO; Carl Roth, Karlsruhe, Germany). The medium of control-treated cells and inhibitor-treated cells contained equal amount of DMSO.

The following plasmids were used: pGL4.20 puro HPV16 LCR (57–59), pGL4.20 puro HPV11 LCR and pGL4.20 puro HPV18 LCR (60), pcDNA3.1 ^(+)^-Luciferase containing luciferase under the control of the CMV promoter (61). For cloning pGL4.20 puro HPV16 LCR, the pGL3 basic LCR16 was restricted with HindIII and the LCR was inserted into the HindIII-restricted target vector pGL4.20 (Promega, Fitchburg, MA, USA). The pGL4.20 puro HPV11 LCR and the pGL4.20 puro HPV18 LCR plasmids were created by cloning HPV11 LCR and HPV18 LCR into pGL4.20 puro using SacI/BglII, respectively. pEGFP-N3 was purchased from Clontech Laboratories, Inc. (Pulo Alto, CA, USA). pEGFP-N1 ErbB2 WT and pEGFP-N1 ErbB2 ΔNLS were kindly provided from Dr. Mien-Chie Hung (University of Texas, Texas, USA) (62). The pcDNA3.1 ^(+)^-ErbB2 plasmid was created by cloning ErbB2 into pcDNA3.1 ^(+)^ backbone (Thermo Fisher Scientific, Frankfurt am Main, Germany). The *erbb2* was amplified from mEmerald-ERBB2-N-18 (Plasmid #62755, from Addgene) using forward primer harboring HindIII restriction site: CACAAAGCTTGCCACC ATGGAGCTGGCGGCCTTGTG and reverse primer containing stop codon and NotI restriction site: GTTAGGTACCTCACACTGGCACGTCCAGACCCA.

### 2.2 Cell lines

Human immortalized keratinocytes (HaCaT) were purchased from Cell Lines Services (CLS; Eppelheim, Germany). The human cervical carcinoma cell line (HeLa) that contains multiple copies of integrated HPV 18 DNA (63) and was purchased from the German Resource Center of Biological Material (DSMZ, Germany). The human cervical carcinoma cell line CaSki contains multiple copies of integrated HPV 16 DNA (64) and was kindly provided by W. Zwerschke, Institute for Biomedical Aging Research, Innsbruck, Austria. The cells were grown at 37°C in Dulbecco’s modified Eagle’s medium (DMEM) (Invitrogen) supplemented with 1% Glutamax (Invitrogen), 10% fetal calf serum (FCS; Biochrom AG, Berlin, Germany), 1% Eagle’s minimum essential medium (MEM) non-essential amino acids (GE Healthcare Life Sciences, Chicago, IL, USA). Normal Human Epidermal keratinocytes (NHEK) were purchased from PromoCell (Heidelberg, Germany) and cultivated according to the manufacturer’s instructions.

### 2.3 Cell binding assay

HaCaT cells were transfected with control siRNA or ErbB2-specific pool of siRNAs. Two days later the cells were detached with 0.05% trypsine/2.5 mM EDTA, resuspended in DMEM and transferred into siliconized reaction tubes to minimize virus binding to the tubes. Control cells (cells transfected with control siRNA) were pre-treated with 80nM polyethylenimine (PEI; Sigma-Aldrich), an inhibitor of HPV binding and infection (65), for 1h at 4°C. Subsequently, the cells (control siRNA-treated with and without PEI, and ErbB2-specific siRNAs-treated) were incubated with ≈300 HPV16 viral genome equivalents (vge) per cell for 1 hour at 4°C on an overhead rotator to prevent virus entry. Afterwards, the cells were extensively washed with phosphate-buffered saline (PBS) to remove unbound pseudovirus and collected in sodium dodecyl sulfate (SDS) sample buffer for Western blotting.

### 2.4 Endosomal preparation and quantitative mass spectrometry

The cells were either left untreated or were exposed to HPV16 PsVs for four or seven hours. Endosomes of noninfected and infected cells were prepared as described previously (66–68). The cells were homogenized and a post-nuclear supernatant was prepared. This supernatant is fractionated by a discontinuous sucrose density gradient and fractions containing early endosomes are collected at the 25%/35% interface. The fractions enriched in early endosomes were identified by immunoblotting using specific endosomal marker, Rab5.

For quantitative mass spectrometry based proteomic analyses, early endosomes were pelleted by ultracentrifugation, proteins were reduced by adding 5 mM DTT, free cysteines alkylated with iodoacetamide (Sigma-Aldrich), and proteins digested with 0.2 μg porcine sequencing grade trypsin (Promega) (69). Nanoscale liquid chromatography of tryptic peptides was performed with a Waters NanoAcquity UPLC system equipped with a 75 μm × 150 mm BEH C18 reversed phase column and a 2.6 μl PEEKSIL-sample loop (SGE, Darmstadt, Germany). Mass spectrometry analysis of tryptic peptides was performed using a Waters Q-TOF Premier API system, operated in V-mode with typical resolving power of at least 10,000. All analyses were performed using positive mode ESI using a NanoLockSpray source. For data processing and protein identification the continuum LCMS^E^ data were processed and searched using the IDENTITY^E^- Algorithm of ProteinLynx Global Server (PLGS) version 2.3. The resulting peptide and protein identifications were evaluated by the software using statistical models similar to those described by Skilling et al. (70). Protein identifications were assigned by searching the UniProtKB/Swiss-Prot Protein Knowledgebase Release 52.3. Identifications were filtered at 1% peptide level FDR based on a 5x randomized decoy database search.

### 2.5 Production of pseudoviruses

HPV16 pseudoviruses (PsVs) were prepared as previously described by Buck and colleagues (71). Expression plasmid carrying codon-optimized L1 and L2 protein was co-transfected with pGL4.20 puro HPV16 LCR promoter reporter plasmid into HEK 293TT cells. Positioning the luciferase gene under the control of the HPV16 LCR, allows studies on transcriptional regulation of LCR and early promoter activity as described previously (59, 72). For visualization of viral DNA by Click it chemistry (Click-iT EdU Alexa Fluor™ 488 Imaging Kit) in STED experiments, HEK293TT cells were treated with the modified thymidine analogue 5-ethynyl-2’deoxyuridine (EdU) during the production of PsVs (59, 71, 73). Two days after transfection the cells were lysed and the PsVs were purified from the cell lysates using OptiPrep (Sigma-Aldrich) gradient centrifugation. Quantification of marker plasmid positive PsVs (viral genome equivalents, vge) per cell was performed by qPCR in an AB 7300 RT-PCR System as described previously (73).

### 2.6 Pseudoinfection assay

The cells were incubated with siRNAs or ErbB2 inhibitors. Subsequently, the cells were exposed to ≈100 (or 500 for NHEK) vge per cell for 24 hours (or 48 hours for NHEK). For inhibition studies, the cells were pre-treated with inhibitor for 1 hour before HPV16 addition and cultured for 24 hours or were infected for 24 hours and treated with inhibitor for five hours. Next, the cells were washed once with 1xPBS and lysed for 20 min on a shaking device using 250 µl per well of 1x Cell Culture Lysis Reagent (Promega). Cell lysates were centrifuged at full speed in a benchtop centrifuge for 3 min and 150 µl of the supernatant was transferred into a 96-well plate for luciferase measurements. The measurement was performed with the Tristar LB 941 luminometer (Berthold Technologies, Bad Wildbad, Germany), which added 50 µl of the luciferase substrate buffer (1 mM coenzyme A, 50 mM luciferin, 50 mM ATP, 0.5 M EDTA, 1 M DTT, 0.5 M Tris-HCl pH 7.8, 1 M MgSO_4_) to each well automatically. Afterwards the plate has been shaken, incubated for 5 sec at room temperature and the luciferase activity was measured for 15 sec. The luciferase activity was normalized to lactate dehydrogenase (LDH) measurements (CytoTox-ONE™ Homogeneous Membrane Integrity Assay, Promega) as a measure for viable cell amounts. LDH activities were measured according to manufacturer’s instructions using the Tristar LB 941 luminometer.

### 2.7 Promoter assay

A Promoter assay with inhibitors was performed in 24-well plate on cells with approximately 60% confluence at the time of transfection with 0.5 µg of pGL4.20 puro HPV16 LCR plasmid using PEI. Plasmid transfection was performed 5 hours prior inhibitor addition. A Promoter assay after ErbB2 depletion was performed in 24-well plate on cells with approximately 60% confluence at the time of transfection. Transfection involved 0.5 µg of pGL4.20 puro HPV11, HPV16, HPV18 LCR or pcDNA3.1 ^(+)^-Luciferase (CMV) plasmid using PEI (58). A Promoter assay after increasing ErbB2 amounts was performed in 24-well plate on cells with approximately 60% confluence at the time of co-transfection with various amounts of pcDNA3.1 ^(+)^-ErbB2 and 0.2 µg of pGL4.20 puro HPV16 LCR plasmid using PEI. A Promoter assay investigating NLS of ErbB2 was performed in 24-well plate on cells with approximately 60% confluence at the time of co-transfection with 0.8 µg of pEGFP-N3, ErbB2 WT or ErbB2 ΔNLS and 0.2 µg of pGL4.20 puro HPV16 LCR plasmid using PEI. All experiments were analyzed 24 hours after plasmid transfection. In all experiments the luciferase counts were normalized to the LDH measurements as mentioned above.

### 2.8 siRNA-mediated knockdown

The following sequences of the ErbB2 siRNAs were used: CAAAGAAAUCUUAGACGAA (#1), CGGCCCUAAGGGAGUGUCUAA (#2), GUGUGCACCGGCACAGACA (#3) and provided by Sigma-Aldrich. ErbB2#pool denotes a mixture of equal amounts of three single ErbB2 siRNAs (used for initial ErbB2 knockdown in pseudoinfection assay of HaCaT cells) or of two single ErbB2, siRNA #1 and #3 (for all other knockdown experiments). AllStars Negative Control siRNA was used as non-silencing control and was obtained from Qiagen (Hilden, Germany). Cells were transfected with 15 nM siRNA for 48 hours using Lipofectamine RNAiMAX (Invitrogen) according to the manufacturer’s instructions.

### 2.9 Reverse transcription quantitative polymerase chain reaction

Gene expression of target genes was determined via Reverse Transcription quantitative Polymerase Chain Reaction (RT-qPCR). CaSki or HeLa cells were treated with the control siRNA or the pool of two ErbB2 siRNAs, siRNA#1 and #3 and 48 h later lysed for RT-PCR. Through the whole experiment the cells were kept in a subconfluent state. Total RNA was extracted from the sample material using QIAGEN RNeasy Isolation Mini Kit (Qiagen) according to the manufacturer’s instructions. The RNAs’ concentration and purity were subsequently analyzed using NanoDrop ND-1000 Spectrophotometer. 1.0 µg of total RNA was further used for reverse transcription. Reverse Transcription was performed using primaReverse RT-KIT, First-Strand Reverse Transcription Kit (Steinbrenner Laborsysteme GmbH, Wiesenbach, Germany) according to the manufacturer’s protocol. Gene expression of target genes was determined using primaQuant CYBR 2x qPCR blue Master Mix with SYBRGreen-low ROX (Steinbrenner) via quantitative Real-Time PCR using Applied Biosystems 7500 Real Time PCR System. As thermal cycle protocol following parameters were used: 95°C for 3 minutes followed by 40 cycles two-step PCR using 95°C for 10 seconds (denaturation) and 60°C for 30 seconds (annealing and elongation) with a fluorescence detection step at the end of each cycle. The expression intensity of each gene of interest was determined using actin as a reference gene. The following primers were used for detection of HPV18 (NC_001357.1) E6 forward (F): GTGCCAGAAACCGTTGAATCC and reverse (R): CGAATGGCACTGGCCTCTAT, for detection of HPV18 E7 (NC_001357.1) F: ACATTTACCAGCCCGACGAG and R: GGTCGTCTGCTGAGCTTTCT, for HPV16 (NC_001526.4) E6 F: AATGTTTCAGGACCCACAGG and R: GTTGCTTGCAGTACACACATTC, for HPV16 E7 F: CAGCTCAGAGGAGGAGGATG and R: CACAACCGAAGCGTAGAGTC, for β-actin, F: TGAAGATCAAGATCATTGCTCCTCC and R: AGAAGCATTTGCGGTGGACGAT.

### 2.10 Confocal- and STED-microscopy

HaCaT cells were plated onto PLL-coated glass-coverslips in 6-well plates. For inhibitor experiments, 24 hours after seeding, the cells were incubated for 1 h with DMSO or tucatinib and subsequently incubated with EdU-PsVs. For ErbB2 knockdown experiments, HaCaTs were incubated for 3 h with EdU-PsVs 48 h after siRNA transfection. Cells were washed in PBS and membrane sheets were generated in ice-cold sonication buffer (120 mM potassium glutamate, 20 mM potassium acetate, 10 mM EGTA, 20 mM HEPES, pH 7.2) as previously described (23). For immunostaining, membrane sheets were fixed in 4% PFA in PBS for 30 min at RT. PFA was removed and residual PFA was quenched using 50 mM NH_4_Cl in PBS for 30 min at RT. Membrane sheets were briefly permeabilized with 0.2% Triton X-100 in PBS for 5 min. Afterwards, the sample was blocked with 3% BSA in PBS for 30 min. Staining of EdU-PsVs was performed by click-labeling of the plasmid DNA with fluorescein for 30 min at RT according to the manufacturer’s instructions (EdU Click 488 kit, Carl Roth, cat# 7773.1). Afterwards, staining with primary rabbit pAb against CD151 and secondary antibody STAR RED goat anti-rabbit was performed in 3% BSA-PBS for 2 hours and 1 hour, respectively, with a washing step in between. Finally, samples were washed and mounted on microscopy slides using ProLong^®^ Gold antifade mounting medium (Invitrogen, cat# P36930).

For confocal and STED microscopy, coverslips were imaged using a 4-channel easy3D super-resolution STED optics module (Abberior Instruments, Goettingen, Germany) combined with an Olympus IX83 confocal microscope (Olympus, Tokyo, Japan), equipped with an UPlanSApo 100 × (1.4 NA) objective (Olympus, Tokyo, Japan) (available at the LIMES institute imaging facility, Bonn, Germany). EdU-PsVs, click-labeled with fluorescein (see above), were excited with a 485 nm laser and fluorescence was recorded at 500–550 nm. STAR RED was excited with a 640 nm laser and detected with a 650–720 nm filter. A pulsed STED laser 775 nm (for STAR RED) was used for depletion of STAR RED. The pinhole size was set to 60 µm. STED micrographs were recorded via a time-gated detection with 0.75 ns delay and 8 ns gate width. Pixel size was set to 25 nm. Per condition and biological replicate, 20 images were recorded.

Image analysis was performed with the program ImageJ. With reference to the confocal- and STED-images of CD151, subsequently recorded in the confocal- and STED-channel, we employed the plugin Align slice (Gabriel Landini, University of Birmingham) to correct the confocal image of the PsVs (simultaneously recorded with the CD151 image) for lateral shifts with respect to the STED-channel. To improve maxima detection and reduce pixel noise, images were smoothed with a Gaussian blur (σ = 1) prior to analysis. Images were further analyzed using a custom written macro (74), detecting local maxima with the ‘Find Maxima’ function. Only maxima brighter than 4 intensity counts were considered. Using another macro (provided by Dominik Sons, LIMES Institute, University of Bonn), the PsV-ROIs generated by the first macro were enlarged from 2 to 18 pixel radial size, and these 37-pixel-diameter circular regions of interest (ROIs) were propagated to the CD151 channel, counting the maxima within this region.

### 2.11 Western blot analysis

Cells were washed with PBS, lysed in SDS sample buffer containing 10% 2-mercaptoethanol and denatured at 95°C for 5 **min**. Equal amounts of protein were loaded on SDS-polyacrylamide gel (SDS–PAGE). Afterwards, the proteins were blotted onto nitrocellulose membrane (GE Healthcare Life Sciences) and blocked with 5% milk powder in phosphate-buffered saline (PBS) containing 0,1% Tween-20 (PBS-T). Afterwards, the membrane was incubated with primary antibody at 4°C overnight, next day washed in PBST and stained with horseradish peroxidase (HRP)-conjugated secondary antibodies for 1 hour at room temperature (RT). For phosphorylated protein studies, the cells were lysed in lysis buffer containing 5 mM Tris-HCl pH 7.4, 1 mM EGTA, 250 mM sucrose and 1% Triton X-100. The lysis buffer was supplemented with cOmplete™ protease (Roche Applied Science, Penzberg, Germany) and phosphatase inhibitor cocktail PhosSTOP (Roche Applied Science). The cells were lysed applying three freeze-thaw cycles (freezing at -80°C and thawing on 4°C) and denatured at 95°C for 5 minutes in SDS sample buffer. Upon SDS-PAGE, the proteins were blotted onto nitrocellulose membrane and blocked with 5% bovine serum albumin (BSA) powder in Tris-buffered saline containing 0,1% Tween-20 (TBS-T). After overnight incubation with primary antibodies, the proteins were detected using HRP-conjugated secondary antibody. Detection was carried out using the Western Lightning Plus ECL detection reagent (PerkinElmer, Waltham, MA, USA). Signals were recorded either by scientific imaging X-ray films for Western Blot detection Super RX-N (Fujifilm, Duesseldorf, Germany) or Amersham Hyperfilm ECL (Cytiva, Marlborough, MA, USA). Densitometric analysis was performed using ImageJ software (http://imagej.nih.gov/ij/).

### 2.12 Statistics

Data analysis was performed using GraphPad Prism 9 for Windows (Version 9.4.1., GraphPad Software, San Diego, California USA, www.graphpad.com.). Afterwards, the distribution of data was tested using Shapiro-Wilk test. If values from two compared groups followed normal distribution (p > 0.05) differences between the groups were analyzed using Welch’s t test. If values from two compared groups were not normally distributed (p > 0.05), they were analyzed using Mann-Whitney test (Wilcoxon rank sum test). Exact p-values are given and stated in the figure legend for each statistical test where the control was compared to tested condition. The “n” (stated in figure legend) denotes the number of data points per group collected from independent biological replicates. Differences between the groups were considered statistically significant when p ≤ 0.05 with the statistical significance marked in the graph (p ≤ 0.05 *, p ≤ 0.01 **, p ≤ 0.001 ***, p > 0.05 ns = not significant). All experiments were repeated independently at least three times if not stated otherwise.

## 3 Results

### 3.1 ErbB2 receptor tyrosine kinase co-enriches with internalized HPV16 pseudoviruses in endosomes

**Figure 1:**
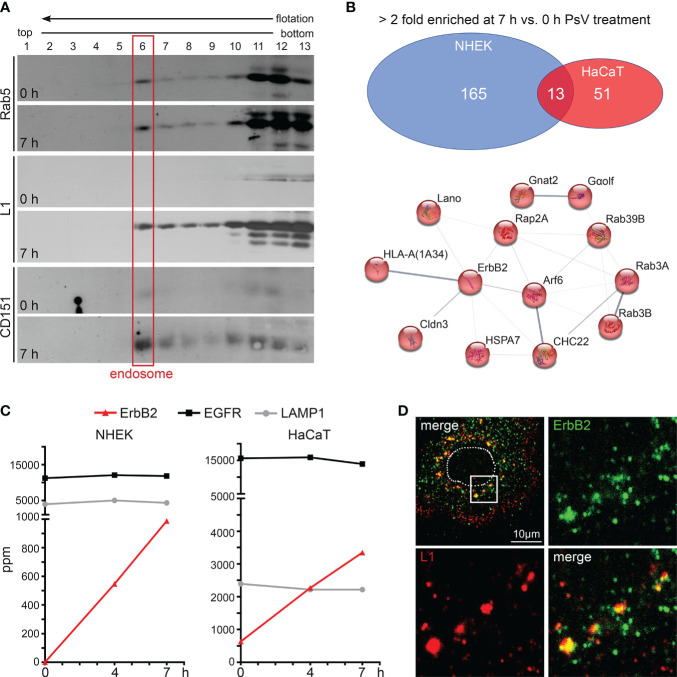
ErbB2 receptor tyrosine kinase enriches with internalized HPV16 PsVs in endosomes. **(A)** Western blot analysis of Rab5, major capsid protein L1 (anti-L1 312F Ab) and CD151 in sucrose flotation density gradient fractions. Rab5 serves as a marker for early endosomes (red frame) and CD151 as positive control for HPV co-internalized receptor component. Note: Rab5 exhibits a dual presence in cytosolic complexes and a membrane-associated state. It is noteworthy that only a small fraction of Rab5, specifically that which is associated with intact endosomes, has the capability to enter the gradient and subsequently accumulates in fraction 6 (endosomes). The remaining Rab5, which does not form complexes with intact endosomes e.g., cytosolic complexes, remains in the loaded post-nuclear supernatant within fractions 11-13. NHEK cells were treated with PsVs for 7 hours (7 h) or left untreated (0 h) and endosomes were isolated by density gradient centrifugation. **(B)** Label-free quantitative mass spectrometry (MS) analysis in NHEK and HaCaT detected enrichment of cellular proteins in endosomal fractions after cells’ exposure to HPV16 PsVs (see also **Supplementary Table S1**). Upper panel: Venn diagram of cellular proteins which were detected as > 2-fold enriched at 7 h compared to the untreated control are displayed for NHEK and HaCaT. Lower panel: String-db analysis (https://string-db.org/) of > 2-fold enriched proteins found in endosomal fractions of both NHEK and HaCaT cells at 7 h when compared to untreated cells: Arf6 (ARF6_HUMAN), Cldn3 (CLD3_HUMAN), CHC22 (CLH2_HUMAN), ErbB2/HER2/neu (ERBB2_HUMAN), Gα_olf_ (GNAL_HUMAN), Gnat2 (GNAT2_HUMAN), HLA-A (1A34_HUMAN), HSPA7/HSP70B (HSP77_HUMAN), Lano/LRRC1 (LRRC1_HUMAN), Rab39B (RB39B_HUMAN), Rab3A (RAB3A_HUMAN), Rab3B (RAB3B_HUMAN) and Rap2A (RAP2A_HUMAN). Lines indicate protein interaction networks with an interaction score of 0.700 (high confidence) for thick lines, 0.400 (medium confidence) for medium lines, and 0.150 (low confidence) for thin lines. **(C)** Graphs with parts per million (ppm) values of total protein for ErbB2 (in red), EGFR (in black), and LAMP1 (in grey) determined by MS of endosomal fractions taken at 0, 4 and 7 h post virus addition (depicted as 0 h, 4 h and 7 h, respectively). ErbB2 is enriched in endosomes during the time course of HPV16 entry in NHEK (left panel) and HaCaT (right panel) cells. As expected, LAMP1 shows comparable endosome content as it is located in all stages of endosomes (76). **(D)** Representative CLSM image of ErbB2 and L1 co-localization in NHEK. Cells were fixed at 7 h and stained with monoclonal anti-ErbB2 antibody (green) and polyclonal rabbit anti-L1 antiserum K75 (red). Nucleus is depicted as a dotted line. Box in the upper left overview indicates the area shown as magnified views.

In target cells, to identify proteins involved in HPV16 entry and infection, we performed proteome analysis of endosomal preparations using normal human epidermal keratinocytes (NHEK) and immortalized keratinocytes (HaCaT) as our model cell lines. The cells were left untreated (0 h) or were treated with HPV16 pseudoviruses (PsVs) for either four or seven hours (4 or 7 h). Endosomes were prepared using flotation density gradient centrifugation in a sucrose step gradient as described previously (66–68, 75). Compared to the neighbored fractions, Western blot analysis shows a clear signal of the endosomal marker in fraction 6, suggesting that this fraction contains the endosomes (**Figure 1A**, red box) (66). After PsV-treatment, the major HPV16 capsid protein L1 is detected in endosomal fractions, confirming successful viral internalization. Additionally, the entry receptor component CD151 showed increased abundance in endosomes upon PsVs addition, validating the co-internalization of the viral receptor complex along with the virus (**Figure 1A**). The endosomal fractions of NHEK and HaCaT cells (0 h, 4 h and 7 h) were subjected to tryptic digestion and quantitative protein analysis by liquid chromatography–mass spectrometry (qLC-MS). Mass spectrometry data (**Supplementary Table S1**) uncovered 178 (NHEK) and 64 (HaCaT) proteins to be more than twofold enriched in endosomes upon HPV16 PsV treatment (7 h vs. 0 h), from which 13 proteins overlap (**Figure 1B**, upper panel), pointing towards a crucial role of these proteins in the context of HPV16 infection. String-db analysis (https://string-db.org/) of these 13 candidates displayed a protein network of plasma membrane proteins and associated factors (GO:0016020: Membrane) with ErbB2 receptor tyrosine kinase as a central component in the network (**Figure 1B**, lower panel). Five of the proteins are involved in signal transduction (ErbB2/HER2/neu, Cldn3, Rap2a, GNAL/Gα_olf_, Lano/LRRC1) (47, 77–79) and five in intracellular trafficking (Arf6, Rab3A, Rab3B, Rab39B, CHC22) (80–83). The enrichment of these proteins suggests that HPV16 particles mediate their recruitment to endocytic pits or endosomes to facilitate subsequent infection steps.

ErbB2 is a central component in the uncovered HPV16/endosome-network (**Figure 1B**) and was also detected previously in endosomal fractions of HeLa cells exposed to HPV16 PsVs (68). While EGFR is also present in high quantities in untreated cells and without any alterations after PsVs treatment, ErbB2 exhibited a substantial PsVs-triggered increase in both NHEK and HaCaT endosomes (**Figure 1C**). This suggests a potential association between ErbB2 and the HPV16 entry receptor complex, or the induction of ErbB2 internalization by virus particles. Moreover, in primary keratinocytes, immunofluorescence analyses revealed strong co-localization between endogenous ErbB2 and the HPV16 major capsid protein L1 (**Figure 1D**). Together, these findings suggest that ErbB2 either may be part of the HPV16 entry receptor complex, play a role in the formation of the entry receptor complex, or just passively co-internalizes.

### 3.2 A strong requirement of ErbB2 for the infection with HPV16 PsVs

**Figure 2:**
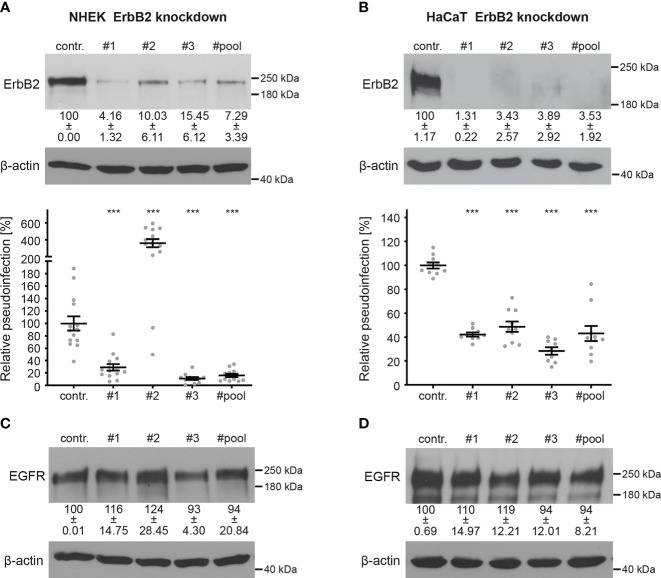
ErbB2 depletion reduces HPV16 PsVs infection. NHEK and HaCaT cells were transfected with control (contr.) or ErbB2 specific siRNA (#1, #2, #3, #pool). Knockdown efficacy was analyzed by Western blot 48 h after siRNA transfection using anti-ErbB2 or EGFR-specific antibody; β-actin was used as loading control. Relative pseudoinfection was assessed by luciferase activity and normalized to lactate dehydrogenase (LDH) activity as cell viability control. Knockdown efficacy and pseudoinfection are given as means ± SEM and the mean for control siRNA-treated cells (contr.) was set to 100%. (**A**, upper panel) Knockdown efficacy of ErbB2 in NHEK using ErbB2-specific siRNAs and a pool of #1 and #3 siRNAs. The statistical difference between the two groups (n = 3 – 4) was analyzed with the Welch’s t test (p < 0.0001 for contr. vs. #1, p = 0.0046 for contr. vs. #2, p = 0.0008 for contr. vs. #3, p = 0.0013 for contr. vs. #pool). (**A**, lower panel) Two days after siRNA transfection NHEKs were infected with HPV16 PsVs for 48 h and analyzed for luciferase counts. The statistical difference between the two groups (n = 11 – 14) was analyzed with the Mann-Whitney test (p = 0.0002 for contr. vs. #2 and p < 0.0001 for contr. vs. #1, contr. vs. #3, contr. vs. #pool). (**B**, upper panel) Knockdown efficacy of ErbB2 in HaCaT using three ErbB2-specific siRNA and a pool thereof. The statistical difference between the two groups (n = 4 – 5) was analyzed with the Mann-Whitney test (p = 0.0159 for contr. vs. #1, contr. vs. #2, contr. vs. #3, contr. vs. #pool). (**B**, lower panel) One day after siRNA transfection HaCaT cells were infected with HPV16 PsVs and 24 h later analyzed for luciferase counts. The statistical difference between the two groups (n = 9 – 10) was analyzed with the Mann-Whitney test (p < 0.0001 for all comparisons (contr. vs. #1, contr. vs. #2, contr. vs. #3, contr. vs. #pool). **(C)** Knockdown efficacy of EGFR in NHEK. For NHEK analysis two biological replicates were used. Values (n = 2) are shown as means ± SD. The statistical difference between the groups (n = 2) was analyzed with the Mann-Whitney test (p= 0.3333 for contr. vs. #1, contr. vs. #2, contr. vs. #3 and p > 0.9999 for contr. vs. #pool). **(D)** Knockdown efficacy of EGFR for HaCaT. The statistical difference between the two groups (n = 4) was analyzed with the Welch’s t test (p = 0.5426 for contr. vs. #1, p = 0.2119 for contr. vs #2, p = 0.6578 for contr. vs #3 and p = 0.5824 for contr. vs #pool). p ≤ 0.001 ***.

To investigate the importance of ErbB2 in HPV16 infection, we conducted PsV infection assays in both ErbB2-depleted and control-treated NHEK and HaCaT cells. Recently developed HPV16 LCR PsVs, in which the LCR of the HPV genome regulates the luciferase reporter gene, allow monitoring specific effects on the early viral gene expression step (59, 72). ErbB2 depletion was achieved using three different specific siRNAs (#1, #2, and #3) (**Figures 2A, B**). In NHEKs, all siRNAs reduced ErbB2 protein levels. The siRNAs #1 and #3 resulted in a significant drop in HPV16 pseudoinfection level of 70% to 90% compared to the control (**Figure 2A**). However, we observed a strong variance in the results obtained with siRNA #2, which displayed a several-fold increase in the HPV16 PsV infection. This suggested that siRNA #2 may induce side effects in NHEK cells, which is why it was excluded from the siRNA pool approach that, indistinguishably from the single siRNAs, reduced expression and pseudoinfection (**Figure 2A**). In HaCaT cells, ErbB2 depletion with all tested siRNAs and the pooled siRNAs showed an essentially complete depletion of ErbB2, accompanied by a 50% to 70% reduction in pseudoinfection level when compared to control siRNA-treated cells (**Figure 2B**). Different cell lines have distinct physiological characteristics, signaling pathways, and expression profiles. Therefore, siRNAs can have different off-target effects in different cell lines. As this might apply to #2, we excluded this siRNA from the siRNA pool used in the following experiments. To further control the specificity of ErbB2 depletion, we examined in both cell lines the level of cellular EGFR, a major interaction partner of ErbB2 (**Figures 2C, D**). The results showed that ErbB2 depletion affected only slightly the expression level of its partner protein.

### 3.3 ErbB2 siRNA decreases levels of phosphorylated Akt and ERK

**Figure 3:**
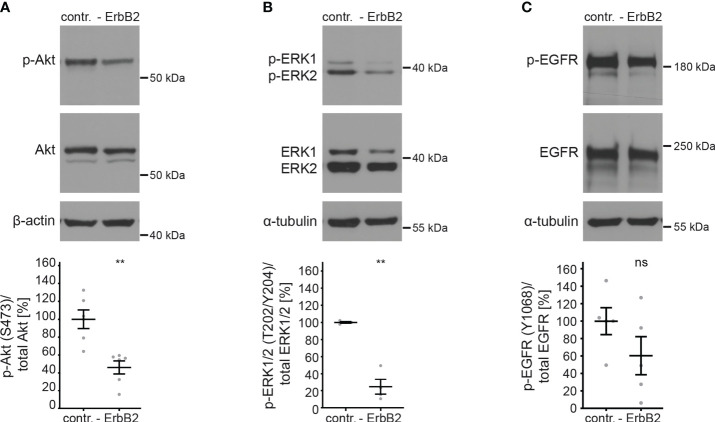
ErbB2 depletion decreases levels of phosphorylated Akt and ERK. HaCaTs were treated with the control siRNA (contr.) or the pool of two ErbB2 siRNAs, siRNA#1 and #3 (- ErbB2) and 48 h later lysed for Western blot (WB) (upper panels). Values are shown as means ± SEM with the mean for control siRNA-treated cells (contr.) set to 100% (lower panels). β-actin or α-tubulin were used as a loading control as indicated. S, T, and Y attached to numbers stand for the phosphorylated (p) amino acids serine, threonine, and tyrosine, respectively. **(A)** WB shows p-Akt and total Akt. The signal was detected with anti-p-Akt (S473) and anti-Akt Abs. Graph displays the ratio of p-Akt to total Akt, related to actin. The statistical difference between the two groups (n = 6) was analyzed with the Mann-Whitney test (p = 0.0022). **(B)** WB shows p-ERK1/2 (T202/Y204) and total ERK1/2. Phosphorylated and total ERK1/2 was detected with anti-p-ERK1/2 (T202/Y204) and anti-ERK1/2 Abs, respectively. Ratio of p-p44/42 (p-ERK) to total p44/42 (ERK) protein, related to tubulin. The statistical difference between the two groups (n = 4) was analyzed with the Welch’s t test (p = 0.0030). **(C)** WB shows p-EGFR (Y1068) and total EGFR. Phosphorylated EGFR was detected using anti-p-EGFR (Y1068) Ab and total EGFR using anti-EGFR Ab. Ratio of p-EGFR (Y1068) to total EGFR protein. The statistical difference between the two groups (n = 5) was analyzed with the Welch’s t test (p = 0.1815). p ≤ 0.01 **, ns (not significant).

To investigate the importance of ErbB2 in the activation of downstream signaling pathways and its involvement in HPV16 entry platform formation, we conducted a series of experiments using HaCaT cells. First, we examined the effect of ErbB2 siRNA on the activation of the above-mentioned signaling pathways Akt and ERK. As expected, the depletion of ErbB2 resulted in a strong and significant decrease in Akt and ERK phosphorylation (**Figures 3A, B**). These findings demonstrate that ErbB2 indeed plays a central role in regulating the activation of these signaling pathways in the HaCaT cell system used in our study.

Next, we investigated whether ErbB2 depletion would affect the phosphorylation of its partner protein EGFR. We observed that the depletion of ErbB2 had a slight and not significant impact on EGFR phosphorylation, as depicted in **Figure 3C**.

### 3.4 ErbB2 is dispensable for HPV16 PsVs binding to the cell-surface and entry-platform formation

**Figure 4:**
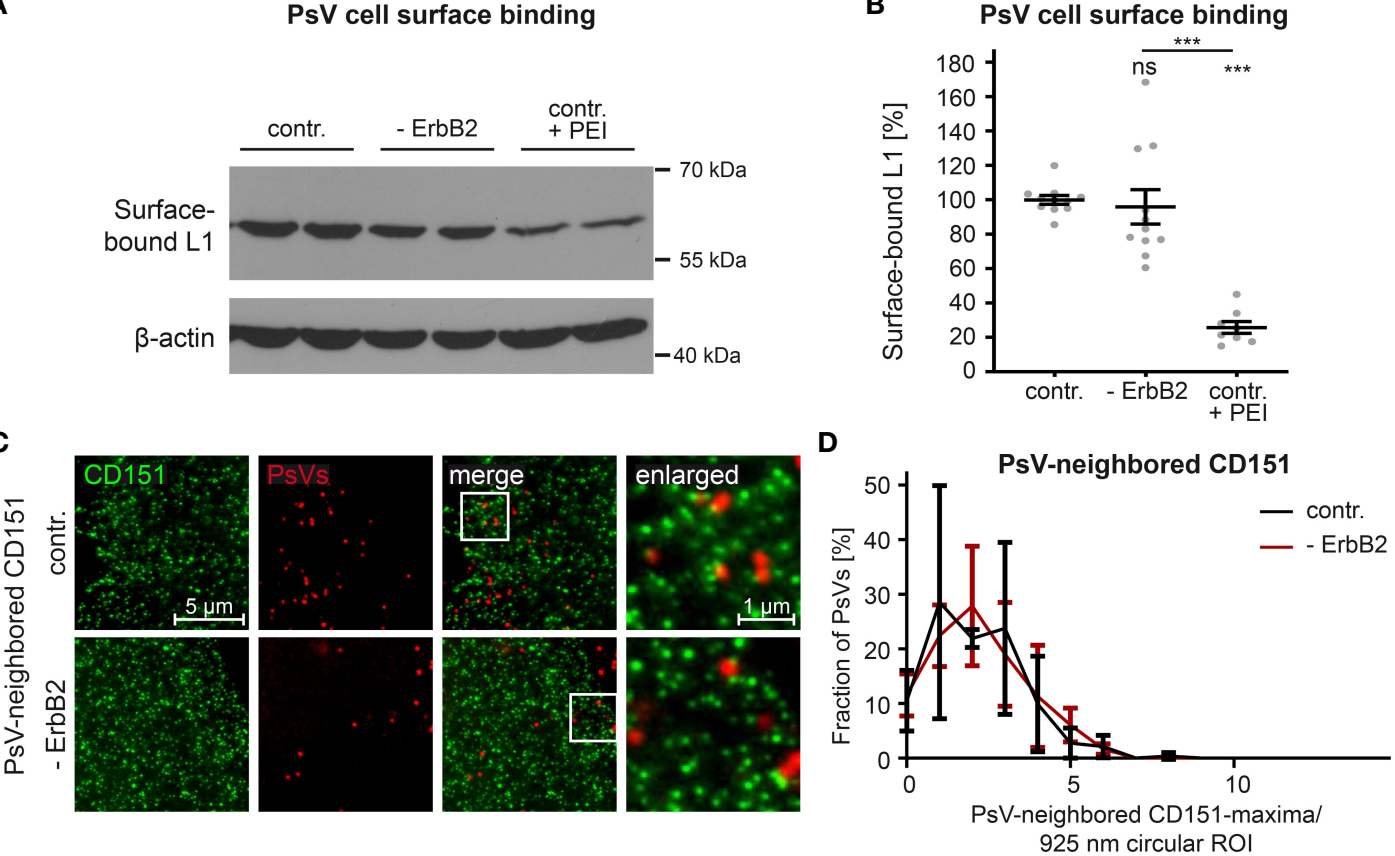
ErbB2 depletion neither affects HPV16 PsVs binding to the cell-surface nor the density of CD151-maxima at PsV-binding sites. **(A, B)** HaCaT cells were treated either with a control siRNA (contr.) or a mixture of two ErbB2-specific siRNAs, siRNA #1 and #3 (- ErbB2). 48 h later control cells were either left untreated or were treated with PEI (contr. + PEI) for 1 h. All cells were exposed to the HPV16 PsVs for 1 h at 4°C to prevent virus endocytosis, washed and processed for Western blot (WB). **(A)** WB showing surface-bound L1 detected using anti-L1 (312-F) Ab. β-actin was used as loading control. **(B)** Quantification of the surface-bound L1 from WBs as shown in **(A)**. Values (n = 8 – 11) are given as means ± SEM and the mean for contr. was set to 100%. The statistical difference between the two groups was analyzed with the Mann-Whitney test (p = 0.1014 for contr. vs. ErbB2; p < 0.0001 for contr. vs. contr. + PEI and ErbB2 vs. contr. + PEI). **(C)** HaCaT cells were transfected either with control or ErbB2 targeting siRNA (- ErbB2) and after 48 h incubated with HPV16 PsVs for 3 h. Then membrane sheets were generated, fixed, permeabilized and stained. EdU-PsVs (red) were visualized by click-labeling of the plasmid DNA in the confocal channel, whereas CD151 (green) was visualized by antibody labelling in the STED-channel. Images from the same channels are scaled equally and are displayed using a linear lookup table. **(D)** An image algorithm detects local maxima in the PsV- images. At the maxima positions of the PsVs, 925 nm diameter circular ROIs (37-pixel-diameter) were placed, in which the number of CD151-maxima was counted. The percentage of PsVs is plotted versus the number of their neighbored CD151-maxima. Values (n = 3) are given as means ± SD. The statistical difference between the two groups was analyzed with the Welch’s t test and showed no significant effect. p ≤ 0.001 ***, ns (not significant).

In order to study the role of ErbB2 in a specific step of HPV infection, we first examined virus binding to the cell surface. Negatively charged heparan and chondroitin sulfate proteoglycans are the major primary attachment factors for HPV (84–88). As a positive control for our experiments, to inhibit HPV-cell binding, we used the polycationic agent polyethyleneimine (PEI), as characterized previously (65). The L1 protein level in PsV-incubated cell lysates was quantitatively analyzed to determine the impact of PEI treatment and ErbB2 depletion on virus attachment. **Figures 4A, B** demonstrate that PEI inhibited PsV-binding by ∼ 80%, validating our experimental setup. However, ErbB2-depleted cells showed equal levels of surface-bound L1 when compared to control siRNA-treated cells. Hence, ErbB2 is dispensable for the primary attachment of HPV16 to the cell surface.

During cell-entry, viruses interact with multiple surface components, likely organized by tetraspanins into platforms (89). The tetraspanin CD151 is relevant in HPV infection, as suggested by the binding of PsVs to CD151-patches (22), that in super-resolution microscopy have a local density of about 4 CD151-maxima per µm^2^ (23). To investigate a role of ErbB2 on CD151-maxima crowding specifically at the PsV-binding site, we employed super-resolution STED microscopy on membrane sheets of HaCaT cells depleted of ErbB2 and incubated for three hours with PsVs prior to membrane sheet generation. Membrane sheets were fixed and immunostained for CD151 and PsVs were visualized by click-chemistry (**Figure 4C**). An image algorithm determines the positions of PsVs, and determines in the CD151 channel the CD151-maxima density at or close to the PsV binding site, by counting the maxima in a 900 nm circular region of interest (ROI) centered at the PsV-binding site. As shown in **Figure 4D**, the neighbored CD151-maxima distribution shows a broad peak at ∼ 2 maxima/ ROI (circle = 0.67 µm^2^), which corresponds to 3 maxima per µm^2^, a value close to the previously reported density of 4 maxima/µm^2^. The data indicate that the absence of ErbB2 has no effect on CD151-maxima crowding at the PsV-binding site, or in other words, on HPV16 entry platform formation.

In conclusion, despite its identified role as a central component in the activation of Akt and ERK pathways in HaCaT cells, ErbB2 or its downstream signaling neither appears to impact HPV16 attachment to the cell-surface, nor is required for regulating the density of CD151-maxima at the PsV-binding site.

### 3.5 ErbB2-targeting inhibitors tucatinib and CP-724714 efficiently block ErbB2, Akt and ERK phosphorylation

**Figure 5:**
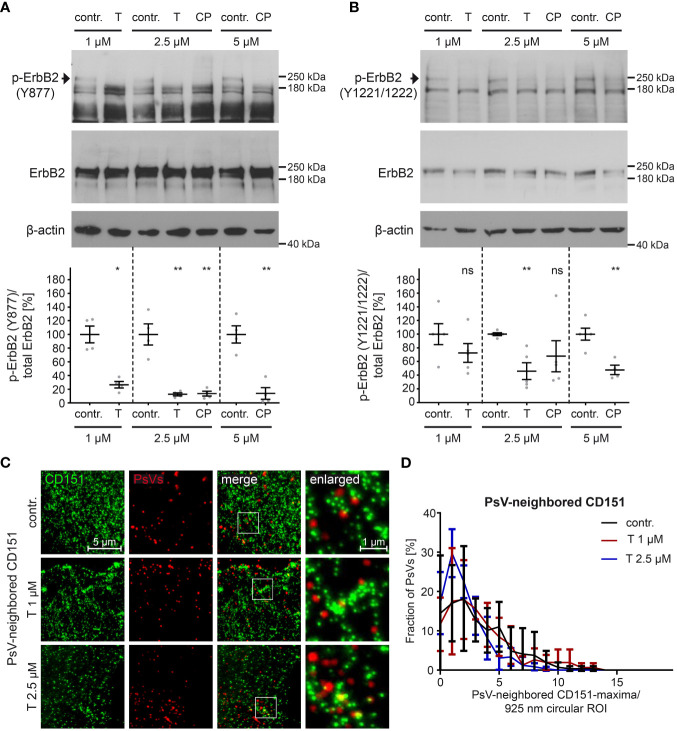
ErbB2-targeting inhibitors tucatinib and CP-724714 inhibit ErbB2 phosphorylation but do not affect the density of CD151-maxima at PsVs binding-sites. **(A, B)** HaCaT cells were treated with different concentration (in µM) of either control (contr.) or ErbB2-targeting inhibitors for 1 h and then processed for Western blot (WB) analysis (upper panels). Contr. stands for control (DMSO solvent; the medium of control-treated cells and inhibitor-treated cells contained equal amount of DMSO), T for tucatinib, CP for CP-724714, and Y for tyrosine phosphorylation sites. Values are shown as means ± SEM with the mean for the contr. set to 100% (lower panels). β-actin was used as a loading control as indicated. **(A)** WBs show p-ErbB2 (Y877) and total ErbB2 for the indicated contr. and inhibitor concentrations. Phosphorylated ErbB2 was detected using anti-p-ErbB2 (Y877) and total ErbB2 with anti-ErbB2 Abs. Ratio of p-ErbB2 (Y877) to total ErbB2, related to actin. The statistical analysis between the two groups of interest (n = 4) was analyzed with the Mann-Whitney test (p = 0.0286 for contr. 1 vs. T 1) and the Welch’s t test (p = 0.0098 for contr. 2.5 vs. T 2.5, p = 0.0090 for contr. 2.5 vs CP 2.5, p = 0.0020 for contr. 5 vs. CP 5). **(B)** WBs show p-ErbB2 (Y1221/1222) and total ErbB2. Phosphorylated ErbB2 was detected using anti-p-ErbB2 (Y1221/1222) and total ErbB2 with anti-ErbB2 Abs. β-actin was used as a loading control. Ratio of p-ErbB2 (Y1221/1222) to total ErbB2, related to actin. The statistical difference between the two groups (n = 4 – 5) was analyzed with the Welch’s t test (p = 0.2172 for contr. 1 vs. T 1, p = 0.0023 for contr. 5 vs. CP 5) and with the Mann-Whitney test (p = 0.0079 for contr. 2.5 vs. T 2.5, p = 0.1058 for contr. 2.5 vs. CP 2.5). **(C)** HaCaT cells were treated with control buffer (contr.) or 1 µM or 2.5 µM of the ErbB2-specific inhibitor tucatinib (T) for 1 h prior to PsVs addition for 3h. Then, membrane sheets were generated, fixed, permeabilized and stained. EdU-PsVs (red) were visualized by click-labeling of the plasmid DNA in the confocal channel, whereas CD151 (green) was visualized by antibody staining in the STED-channel. Images from the same channels are scaled equally and are displayed using a linear lookup table. **(D)** An image algorithm detects local maxima in the PsV- images. At the maxima positions of the PsVs, 925 nm diameter circular ROIs (37-pixel-diameter) were placed, in which the number of CD151-maxima was counted. The percentage of PsVs is plotted versus the number of their neighbored CD151-maxima. Values (n = 3) are given as means ± SD. The statistical difference between the two groups was analyzed with the Welch’s t test and showed no significant effect. p ≤ 0.05 *, p ≤ 0.01 **, ns (not significant).

To assess more thoroughly the efficacy of pharmacological inhibitors in targeting ErbB2 and its downstream signaling pathways in HaCaT cells, we tested different concentrations of tucatinib and CP-724714. These inhibitors offer certain advantages over siRNAs, as they preserve the target molecule and allow for better temporal resolution of cellular processes. First, we determined concentrations of tucatinib and CP-724714 that effectively block ErbB2 phosphorylation (**Figures 5A, B**). Specifically, we focused on the phosphorylation of three key tyrosine (Y) residues, Y877 and Y1221/1222, involved in ErbB2 autophosphorylation (90, 91). The inhibitors efficiently prevented the phosphorylation of Y877 at all concentrations tested (**Figure 5A**). Furthermore, significant inhibition of phosphorylation at the Y1221/1222 sites was observed with 2.5 µM tucatinib and 5 µM CP-724714 (**Figure 5B**).

We further tested whether tucatinib has any effect on the CD151-maxima density at PsV-binding sites, which was not the case (**Figures 5C, D**). Hence, in line with the siRNA knockdown (**Figure 4D**), the ErbB2 activation has no role in regulating this process.

**Figure 6:**
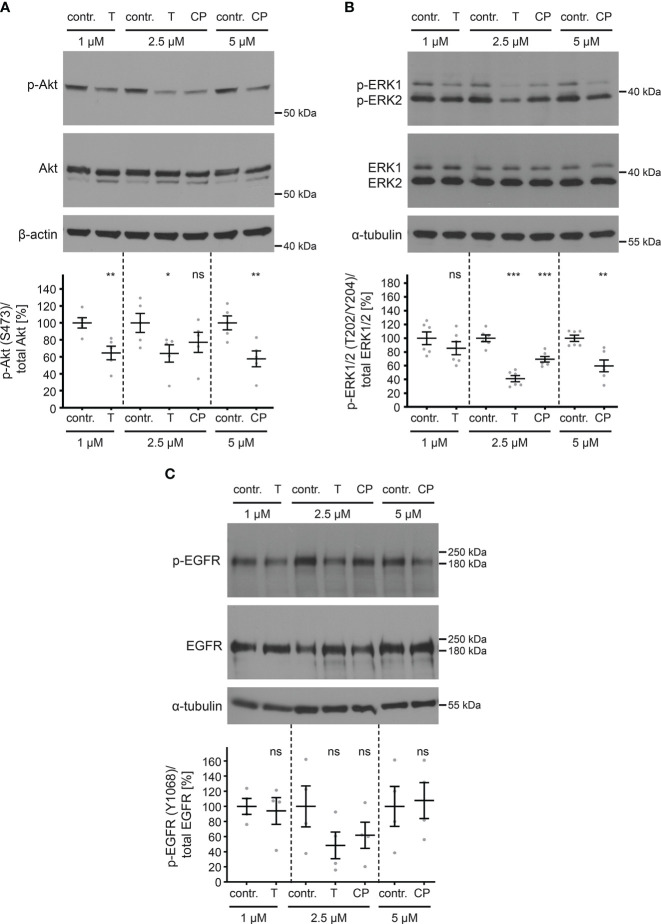
ErbB2 inhibition decreases levels of phosphorylated Akt and ERK but not phosphorylated EGFR. HaCaT cells were treated for 1 h with different concentration (in µM) of either control (contr.) or ErbB2-targeting inhibitors and analyzed by Western blot (WB) (upper panels). Contr. stands for control (DMSO, solvent; the medium of control-treated cells and inhibitor-treated cells contained equal amount of DMSO), T for tucatinib, and CP for CP-724714. Values are shown as means ± SEM with the mean for control set to 100% (lower panels). β-actin or α-tubulin were used as a loading control as indicated. **(A)** WBs show p-Akt and total Akt. The signal was detected with anti-p-Akt and anti-Akt Abs. Ratio of p-Akt to total Akt protein, related to actin. The statistical analysis for the two groups of interest (n = 5) was analyzed with the Welch’s t test (p = 0.0088 for contr. 1 vs. T 1, p = 0.0459 for contr. 2.5 vs. T 2.5, p = 0.1989 for contr. 2.5 vs. CP 2.5, p = 0.0097 for contr. 5 vs. CP 5). **(B)** WBs show p-ERK1/2 and total ERK1/2. The signal was detected with anti-pERK1/2 and anti-ERK1/2 Abs. Ratio of p-ERK1/2 to total ERK1/2 protein, related to tubulin. The statistical analysis for the two groups of interest (n = 6) was analyzed with Welch’s t test (p = 0.3037 for contr. 1 vs. T 1, p < 0.0001 for contr. 2.5 vs. T 2.5 and p = 0.0009 for contr. 2.5 vs. CP 2.5) and Mann-Whitney test (p = 0.0022 for contr. 5 vs. CP 5). **(C)** WBs show p-EGFR (Y1068) and total EGFR. Phosphorylated EGFR was detected using anti-p-EGFR (Y1068) and total EGFR with anti-EGFR Abs. Ratio of p-EGFR (Y1068) to total EGFR, related to tubulin. The statistical difference between the two groups (n = 4) was analyzed with the Welch’s t test (p = 0.7848 for contr. 1 vs. T 1, p = 0.1686 for contr. 2.5 vs. T 2.5, p = 0.2869 for contr. 2.5 vs. CP 2.5, p = 0.8349 for contr. 5 vs. CP 5). p ≤ 0.05 *, p ≤ 0.01 **, p ≤ 0.001 ***, ns (not significant).

Having established the efficacy of the inhibitors in blocking ErbB2 phosphorylation, we next investigated their effects on downstream signaling through the Akt and ERK pathways. Both tucatinib and CP-724714 led to a decrease in phosphorylated Akt and ERK proteins (**Figures 6A, B**). EGFR phosphorylation on Y1068 was shown to be linked to Akt and ERK activation (92–94). Notably, the phosphorylation of EGFR was only slightly, but not significantly affected by the treatment with all tested concentrations of inhibitors (**Figure 6C**).

This demonstrates that ErbB2 specific TKIs are useful tools in blocking not only ErbB2 activation but also downstream signaling in HaCaT cells with only slightly affecting EGFR.

### 3.6 ErbB2 regulates HPV promoter activity and gene expression

**Figure 7:**
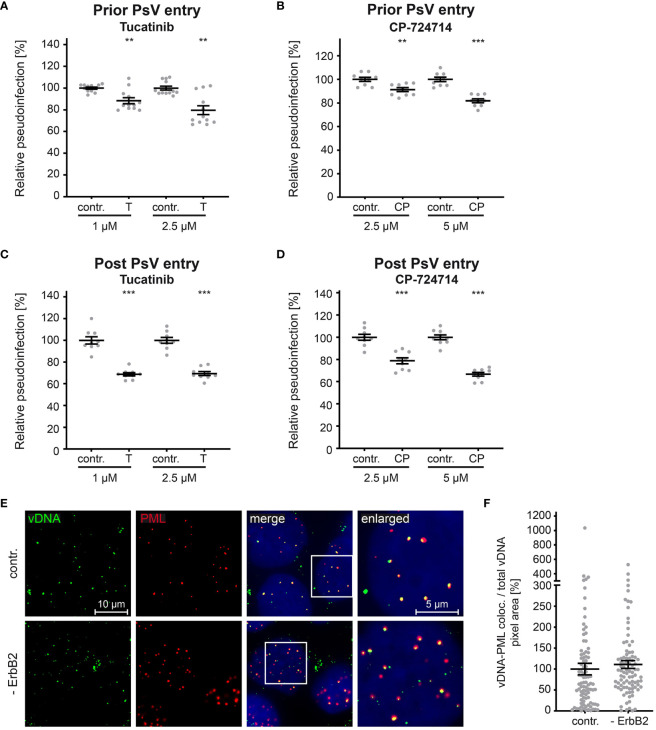
ErbB2 affects post entry steps of HPV PsVs infection. (A, B) HaCaT cells were treated with solvent control (contr.) or the indicated concentration (in mM) of tucatinib (T) or CP-724714 (CP) for 1 h and subsequently exposed to HPV16 PsVs. The luciferase and the LDH activities were assessed 24 h later. **(A)** Effect of tucatinib. The statistical difference between the two groups (n = 12) was analyzed with the Mann-Whitney test (p = 0.0042 for contr. 1 vs. T 1 and contr. 2.5 and T 2.5). **(B)** Effect of CP-724714. The statistical difference between the two groups (n = 9) was analyzed with the Welch’s t test (p = 0.0025 for contr. 2.5 and CP 2.5; p < 0.0001 for contr. 5 and CP 5). **(C, D)** HaCaTs were infected with HPV16 PsVs and 24 h later treated with control (contr.) or the indicated concentration of tucatinib or CP-724714 for another 5 h to enable mRNA and protein turn-over after ErbB2 signaling inhibition. **(C)** Effect of tucatinib. The statistical difference between the two groups (n = 9) was analyzed with the Welch’s t test (p < 0.0001 for contr. 1 vs. T 1 and contr. 2.5 vs. T 2.5). **(D)** Effect of CP-724714. The statistical difference between the two groups of interest (n = 9) was analyzed with the Welch’s t test (p < 0.0001 for contr. 2.5 vs. CP 2.5 and contr. 5 vs. CP 5). Relative pseudoinfection was normalized to LDH. Data are given as means ± SEM, and the mean for contr.-treated cells set to 100%. **(E)** Representative images of HaCaT cells transfected either with control (contr.) or two ErbB2 targeting siRNA, siRNA#1 and #3 (- ErbB2) and after 48 h incubated with HPV16 PsVs for 24 h. EdU-PsVs (green) were visualized by click-labeling of the plasmid DNA, whereas PML (red) with anti-PML antibody. Image acquisition was performed using a Zeiss Axiovert 200 M microscope fitted with a Plan-Apochromat 100Å~/1.4 Oil objective (Carl Zeiss, Jena, Germany). Quantification of colocalization was performed by analysis of at least 20 pictures per group using Colocalization Software 4.7 (Carl Zeiss). **(F)** Relative colocalization of vDNA and PML. vDNA pixels colocalizing with PML pixels are given as means ± SEM, and the mean for control siRNA-treated cells (contr.) was set to 100%. p ≤ 0.01 **, p ≤ 0.001 ***.

As previously shown for siRNA-mediated knockdown and tucatinib treatment (**Figures 4**, **5**), our experiments support the conclusion that ErbB2 is dispensable for the initial stages of infection such as binding and entry platform formation. To substantiate this assumption, we performed time-of-addition experiments with the ErbB2 inhibitors. In line with the idea that ErbB2 is dispensable in early steps, inhibitor treatment of cells prior to PsV addition resulted in a weaker effect on relative pseudoinfection level compared to treatment one day post PsV addition (**Figures 7A–D**). Importantly, inhibitors still have an effect at a time point PsV entry is largely completed (post entry) as shown in **Figures 7B, D**. This supports that ErbB2’s major role is late in the infection cascade, such as the viral gene transcription regulated by the long control region (LCR) of HPV16, which occurs after the virus has reached the host cell nucleus. This notion was verified by assessing colocalization of the viral DNA (vDNA) and promyelocytic leukemia nuclear bodies (PML NBs). Quantitative co-immunofluorescence analysis revealed that the level of vDNA-PML signal overlap was not reduced upon ErbB2 depletion (**Figures 7E, F**).

**Figure 8:**
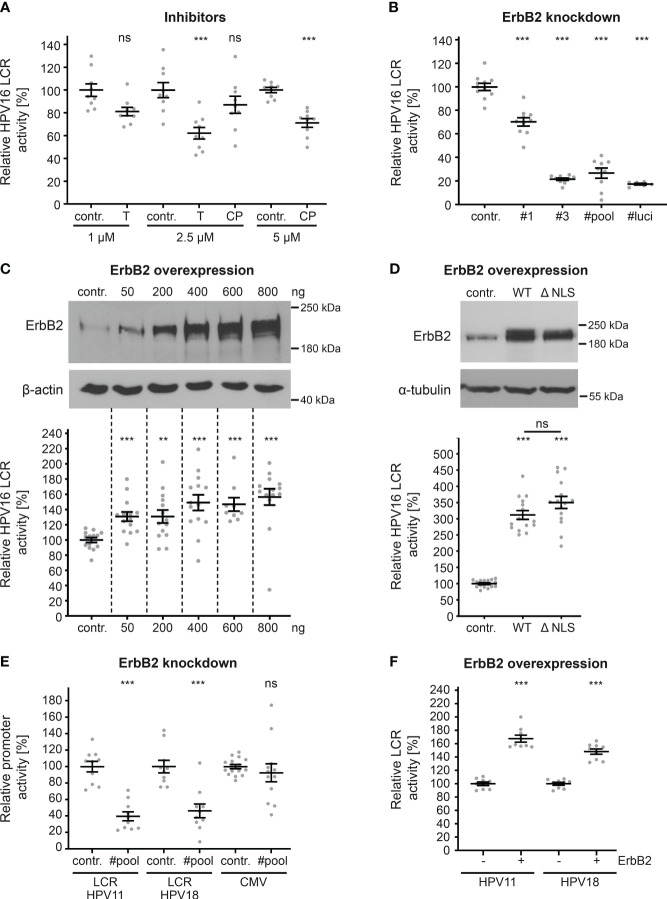
ErbB2 activity influences HPV LCR activity. **(A)** HaCaTs were transfected with HPV16 LCR-harboring pGL4.20 plasmid and treated with solvent control (contr.) or the indicated concentration (in µM) of inhibitor tucatinib (T) or CP-724714 (CP) for another 18 h when luciferase counts were assessed. The statistical difference between the two groups of interest (n = 9) was analyzed with Welch’s t test (p = 0.0141 for contr. 1 vs. T 1, p = 0.0004 for contr. 2.5 vs. T 2.5 p = 0.2175 for contr. 2.5 vs. CP 2.5, p < 0.0001 for contr. 5 vs. CP 5). **(B)** HaCaT cells were treated with control (contr.) or two ErbB2-targeting siRNA (siRNA#1 and #3) and the following day transfected with pGL4.20 plasmid carrying luciferase under the activity of HPV16 LCR. The luciferase and the LDH activities were assessed 24 h after. The #luci denotes luciferase-targeting siRNA. The statistical differences between the two groups (n = 6 - 11) was analyzed with Welch’s t test (p < 0.0001 for contr. vs. #1, contr. vs. #3, contr. vs. #pool, contr. vs. #luci). (**C**, upper panels) HaCaTs were transfected with different amounts of pcDNA3.1 (+)-ErbB2 or left untreated (contr.). The cells were analyzed by Western blot 24 h later. (**C**, lower panel) HaCaTs were co-transfected with HPV16 LCR-harboring pGL4.20 plasmid and different amounts of pcDNA3.1 (+)-ErbB2 or no plasmid (contr.). Promoter activity was assessed 24 h after transfection. The statistical difference between the two groups (n = 9 – 14) was analyzed with the Mann-Whitney test (p < 0.0001 for contr. vs. 50 ng, contr. vs. 600 ng, and contr. vs. 800 ng, p = 0.0034 for contr. vs. 200 ng, p = 0.0002 for contr. vs. 400 ng of ErbB2-harbouring plasmid. (**D**, upper panels) Western blot of HaCaT cells were transfected either with pEGFP-N3 (contr.), pEGFP-N1 ErbB2 WT(WT) or ErbB2 ΔNLS (ΔNLS) expression plasmids. The cells were analyzed by Western blot 24 h later to determine expression levels. (**D**, lower panel) HaCaT cells were co-transfected with HPV16 LCR-harboring pGL4.20 plasmid and either control plasmid pEGFP-N3 (contr.), ErbB2 WT- or ErbB2 ΔNLS-carrying plasmids. Promoter assay was measured 24 h after plasmid transfection. The luciferase counts were normalized to the transfection efficacy calculated from WB bands. The statistical difference between the two groups (n = 15) was analyzed using the Welch’s t test (p < 0.0001 for contr. vs. WT and contr. vs. ΔNLS, p = 0.1072 for WT vs. ΔNLS). **(E)** HaCaTs were transfected either with a control (contr.) or a pool of two ErbB2-targeting siRNAs (#pool). One day later the cells were transfected with a pGL4.20 plasmid carrying HPV11 (LCR HPV11) or HPV18 LCR (LCR HPV18), or with a pcDNA3.1 ^(+)^-Luciferase plasmid (CMV). Comparison for two groups of interest (n = 10 - 16) was analyzed with the Welch’s t test (p < 0.0001 for contr. vs. #pool for LCR HPV11, p = 0.0001 for contr. vs. #pool for LCR HPV18, and p = 0.5172 for contr. vs. #pool for CMV). **(F)** A plus (+) denotes HaCaT cell co-transfected with (800 ng) ErbB2-expression vector pcDNA3.1 ^(+)^ and either HPV11 or HPV18 LCR-harboring pGL4.20 plasmid. A minus (-) denotes HaCaT cells co-transfected with pcDNA3.1 ^(+)^ plasmid (800 ng) and either HPV11 or HPV18 LCR-harboring pGL4.20 plasmid. The statistical difference between the two groups (n = 8) was analyzed with the Mann-Whitney test (p < 0.0001 for - ErbB2 vs. + ErbB2 for HPV11) and Welch’s t test (p < 0.0001 for - ErbB2 vs. + ErbB2 for HPV18). **(A–F)** Luciferase counts were normalized to LDH. Values are given as means ± SEM, and the mean for control-treated cells set to 100%. ErbB2 was detected using anti-ErbB2 Ab. β-actin or α-tubulin were used as a loading control as indicated. p ≤ 0.01 **, p ≤ 0.001 ***, ns (not significant).

Hence, transfection of HaCaT cells with pGL4.20 HPV16 LCR (58, 59) allows us to directly measure the effect of ErbB2 inhibition on the activity of the HPV16 LCR. To inhibit ErbB2, we used concentrations of up to 2.5 and 5 µM of tucatinib and CP-724714, respectively, which show specific effects on ErbB2, Akt, and ERK phosphorylation, as well as on HPV pseudoinfection (**Figures 6**, **7**). The results revealed that both tucatinib and CP-724714 significantly reduced LCR activity by 30-40% compared to the control (**Figure 8A**). Next, we directly assessed the impact of ErbB2 expression levels on LCR promoter activity. ErbB2-depleted cells, achieved through transfection with ErbB2-specific siRNA and subsequently transfected with the HPV16 LCR-containing pGL4.20 plasmid, exhibited a decrease in LCR activity (**Figure 8B**). This is equal to the internal positive control, the luciferase specific siRNA (#luci) that directly targeted the mRNA of the reporter gene. These data demonstrate the strong dependence of the HPV16 LCR activity on ErbB2. Conversely, ErbB2 overexpression resulted in a significant increase in LCR activity, indicating a concentration-dependent effect. When the highest concentration of ErbB2-carrying plasmid was used, the promoter activity increased by 60% (**Figure 8C**).

As previously demonstrated (39, 40, 43, 44, 62), ErbB2’s nuclear translocation can result in direct transcriptional activation. In order to investigate this phenomenon within the context of HPV, we transfected cells with either control, ErbB2 wild-type (WT) plasmid or a plasmid coding for a ErbB2 protein lacking nuclear localization signal (ΔNLS). Interestingly, there was no impact of the ErbB2 nuclear translocation signal, as both ErbB2 constructs, ErbB2WT and the truncated ErbB2 (ΔNLS), exhibited a significant increase in LCR activity, without a significant difference between them (**Figure 8D**). Interestingly, we observed a higher increase on relative LCR activity when pEGFP-ErbB2 WT or ΔNLS was used for overexpression when compared to pcDNA3.1 (+)-ErbB2. Although an equivalent quantity of LCR-luciferase plasmid was transfected, the luciferase counts obtained for the control in **Figure 8C** were approximately 20-fold higher than those for control in **Figure 8D**, the latter of which resulted from co-transfection with the control plasmid pEGFP-N3. The lower baseline reference value in control shown in **Figure 8D** provides an explanation for the observed higher fold changes following ErbB2 overexpression.

Next, we extended our investigation to include promoter sequences of other papillomavirus types (LCR HPV11, LCR HPV18) and cytomegalovirus (CMV) (**Figure 8E**). ErbB2-depleted cells transfected with plasmids containing the LCRs of low-risk HPV11 or high-risk HPV18 exhibited a significant reduction in LCR activity. Moreover, we found that the ErbB2 depletion does not affect CMV promoter-controlled luciferase expression which is an important control for unaffected plasmid delivery, translation and luciferase activity by the treatment. Furthermore, ErbB2 overexpression significantly increased LCR activity for both HPV types (**Figure 8F**). The comparable level of decrease after ErbB2 silencing and increase after its overexpression in promoter activity among HPV11, HPV16, and HPV18 suggests that ErbB2 has a similar impact on the LCRs of all tested HPV types in this study.

**Figure 9:**
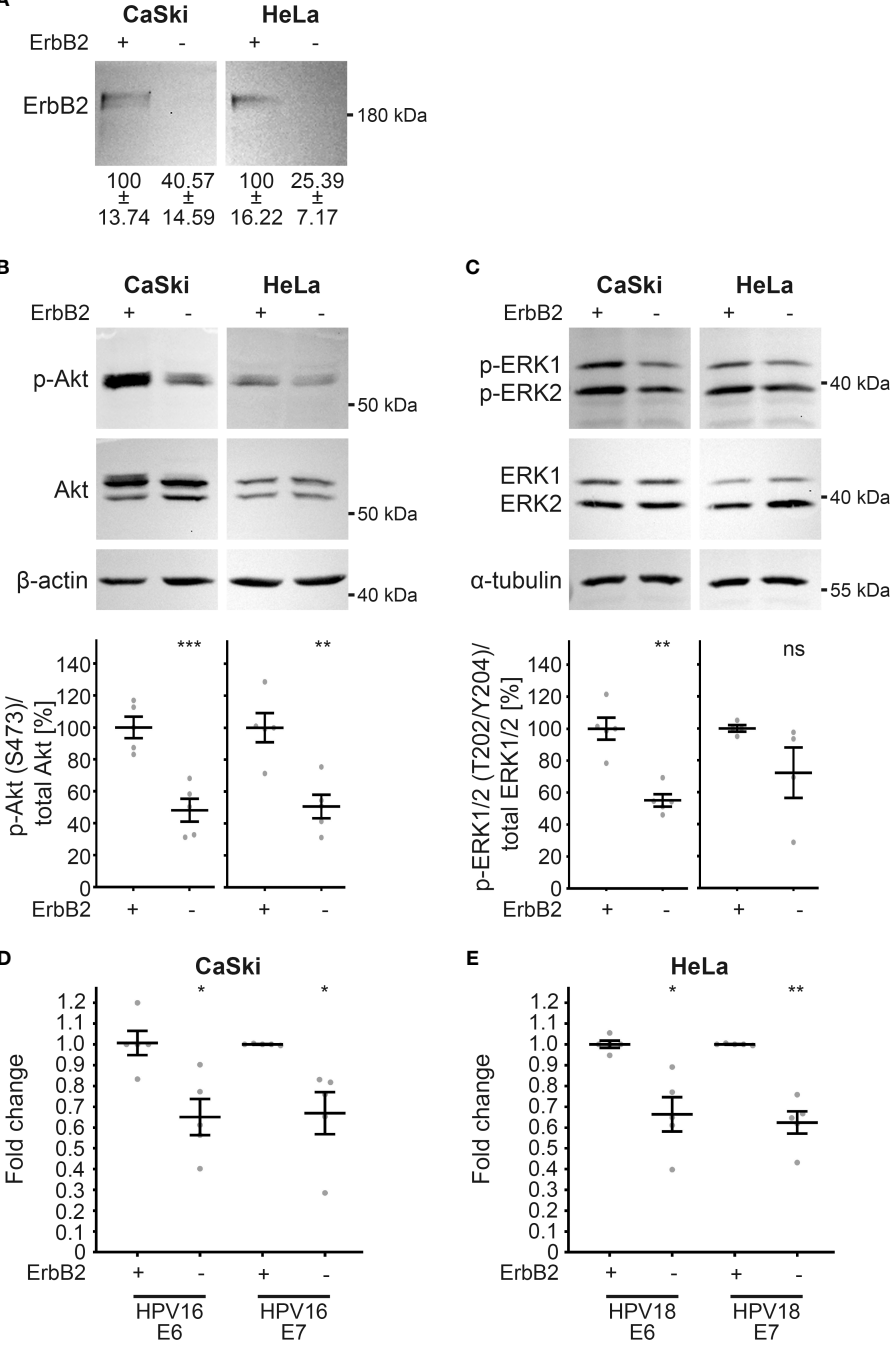
Reduction in Akt and ERK activation upon ErbB2 depletion in CaSki and HeLa cells accompanies reduction in E6 and E7 expression. CaSki or HeLa cells were treated with the control siRNA (+) or the pool of two ErbB2 siRNAs, siRNA#1 and #3 (-) and 48 h later lysed for Western blot (WB) **(A–C)** or RT-PCR **(D, E)**. Through the whole experiment the cells were kept in a subconfluent state. **(A–C)** WBs performed on CaSki (left panel) and HeLa cells (right panels). Values are shown as means ± SEM with the mean for control siRNA-treated cells set to 100%. β-actin or α-tubulin were used as a loading control. Image acquisition was performed using iBright™ CL1500 Imaging System (Thermo Fisher Scientific). **(A)** WB shows total ErbB2. Graph displays the ratio of ErbB2 relative to β-actin (corresponding β-actin WB is shown in **(B)**. The statistical difference between the two groups (n = 5) was analyzed with the Welch’s t test (p = 0.0181 for CaSki and p = 0.0068 for HeLa). **(B)** WB shows p-Akt and total Akt. The signal was detected with anti-p-Akt (S473) and anti-Akt Abs. Graph displays the ratio of p-Akt to total Akt, related to actin. The statistical difference between the two groups (n = 5) was analyzed with the Welch’s t test (p = 0.0007 for CaSki and p = 0.0032 for HeLa). **(C)** WB shows p-ERK1/2 (T202/Y204) and total ERK ½. Phosphorylated and total ERK1/2 was detected with anti-p-ERK1/2 (T202/Y204) and anti-ERK1/2 Abs, respectively. Ratio of p-p44/42 (p-ERK) to total p44/42 (ERK) protein, related to α-tubulin. The statistical difference between the two groups (n = 5 for CaSki and n = 4 for HeLa) was analyzed with the Welch’s t test (p = 0.0010 for CaSki and p = 0.1766 for HeLa). **(D, E)** The expression of viral oncogenes E6 and E7 determined by RT-PCR is shown as a fold change relative to β-actin expression levels. Values are shown as means ± SEM with the mean for control siRNA-treated cells set to 1. **(D)** The fold expression of E6 and E7 in CaSki cells. The statistical difference between the two groups (n = 5) was analyzed with Welch’s t test (p = 0.0112 for E6 and p = 0.0305 for E7). Shown are five biological replicates. **(E)** The fold expression of E6 and E7 in HeLa cells. The statistical difference between the two groups (n = 5) was analyzed with Welch’s t test (p = 0.0140 for E6 and p = 0.0022 for E7). Shown are five biological replicates. p ≤ 0.05 *, p ≤ 0.01 **, p ≤ 0.001 ***, ns (not significant).

To validate the observations made in HaCaT cells, we extended our investigation to well-established HPV16- and HPV18-transformed cell lines, namely CaSki and HeLa, respectively. These cell lines, characterized by the integration of viral DNA into the human genome, exhibit persistent expression of viral oncogenes E6 and E7 (63, 64, 95). Upon ErbB2 depletion (**Figure 9A**), as demonstrated through Western blot, a compelling association with ErbB2-dependent modulation of the Akt and ERK pathways was revealed in these HPV-transformed cervical cancer cell lines. Notably, ErbB2 siRNAs induced a substantial decrease in pAkt and pERK1/2 signals, indicating the pivotal role of ErbB2 in these signaling cascades (**Figures 9B, C**). Concomitant with the attenuation of Akt and ERK activities, a significant reduction in the expression of viral oncogenes E6 and E7 from both HPV types was observed (**Figures 9D, E**). This collective evidence underscores the critical involvement of ErbB2 in sustaining the activity of the Akt and ERK pathways, thereby influencing the expression of key viral oncogenes in HPV-transformed cell lines.

Together, these findings demonstrate that ErbB2 plays a role in mediating the activation of HPV LCRs and their promoters through classical signal transduction pathways, and that inhibitors as well as ErbB2 depletion are useful tools for repressing LCR activity. The observed effect on promoter activity was independent of ErbB2’s nuclear translocation. Additionally, our data indicate that ErbB2-mediated signaling has a general function in regulating promoter activity of various human papillomavirus types, which is essential for efficient gene expression.

## 4 Discussion

In HPV16 PsV transport endosomes, we have uncovered a network of proteins known to mediate trafficking and signaling, with ErbB2 as one of the central components. We demonstrate that the ErbB2 receptor tyrosine kinase, also known as HER2/neu, plays a crucial role in HPV16 infection. ErbB2 depletion significantly reduces HPV16 PsV infection in both NHEK and HaCaT cells, highlighting its potential as a target for further investigation and interventions in the context of HPV16 infection. While ErbB2 is neither involved in the primary attachment and the formation of HPV16 entry platform nor in the delivery of the viral genome to PML bodies, it plays a major role in regulation of the LCR and the incorporated early promoter. The findings also suggest a general function of ErbB2-mediated Akt and ERK signaling on the early promoter of various papillomavirus types. Further analyses on HPV16- and HPV18-transformed cell lines, CaSki and HeLa, showed that ErbB2-mediated Akt and ERK signaling facilitates expression of the viral oncogenes E6 and E7, confirming its crucial role in efficient promoter activity.

The results of the proteome analysis provide valuable insights into the cellular proteins and pathways associated with HPV16 entry and infection, with ErbB2 emerging as a potential player in facilitating viral infection. The study also reveals a network of transmembrane proteins and associated cytosolic factors which might be involved in endocytosis and vesicle trafficking of HPV in primary and immortalized keratinocytes. The enrichment of specific proteins, such as Arf6, and Rab proteins suggests that HPV16 particles stimulate their recruitment to endocytic pits or endosomes to enable virus endocytosis and transport towards the nucleus. Arf6 has already been uncovered to facilitate HPV16 entry (14) which supports the relevance of our screening approach. While the role of multiple Rab proteins has already been analyzed, neither Rab3A/B nor Rab39B have been described in the context of HPV infections (96). Rab3A plays a central role in regulating exocytosis and Rab39B is involved in autophagy (80, 97, 98). Therefore, it is tempting to speculate that these proteins might exert an anti-viral effect by outward transporting or sorting the virus to autophagosomes for degradation. The potential functions of these candidates will be further explored in follow-up studies.

Although growth factor receptors, α6 integrin and CD151 are components of the HPV16 entry receptor complex (16, 18, 19, 21–23), EGFR and α6 integrin are found to be not or only slightly enriched with the virus in endosomes. Likely, the high abundance of both proteins in PsVs-untreated endosomes camouflages its increase upon cell exposure to PsVs. CD151, while not enriched in HaCaTs, is strongly co-enriched with PsVs in NHEK cells, as also demonstrated by Western blot analysis of the gradient fractions, supporting its position within the HPV16 receptor complex in primary keratinocytes. In both preparations the ErbB3 was not detectable. While ErbB4 was initially increased (at 4 h of PsV treatment), the protein amounts decreased within the following three hours. From the ErbB family, only ErbB2 continuously increased in the time course of infection along with the virus in both keratinocyte cell systems. This substantial enrichment detected also in HeLa cells during the time course of HPV16 infection (68) as well as the strong overlap of ErbB2 with L1 signal reinforces ErbB2’s significance in HPV16 infection.

In both keratinocyte cell types, infection assays using HPV16 PsVs carrying a promoter reporter plasmid with the viral LCR as regulatory element, showed strong reduction in pseudoinfection upon ErbB2 depletion. These data implicate the requirement of ErbB2 for the early steps of infection with putative roles in virus binding, entry platform formation, intracellular trafficking or gene expression. Contrary to the initial assumption, ErbB2 was found not to be involved in virus cell binding or entry platform formation. Therefore, it seems plausible that entry platform formation is rather influenced by growth factor availability which facilitates interaction between the virus and receptor tyrosine kinases (RTKs) as suggested earlier (16, 18, 20, 24, 99), than by the presence of ErbB2 or ErbB2’s signal transduction. At the moment, we can only speculate why ErbB2 is routed to endosomes upon HPV entry without playing a role during this process. On the one hand, ErbB2 might be co-internalized upon HPV16 endocytosis via direct interaction with CD151 or EGFR as useless component. On the other hand, ErbB2 might be replaced by EGFR or ErbB4 in ErbB2-inhibited or -depleted cells to complete the entry platform. In addition, ErbB2 might be co-transported into the nucleus within virus-transport vesicles to support gene expression. Comparable to earlier results on HPV16 entry and our results shown here, EGFR-induced Akt signaling facilitates the infectious virus uptake of Influenza A virus (IAV), while ErbB2 inhibitor tucatinib showed no effect on IAV entry but induced a decrease of virus replication (100, 101).

The temporal resolution enabled by pharmacological inhibition has helped to elucidate the role of ErbB2 in different stages of the viral replication cycle. The observation that the inhibitors induced a stronger infection inhibition when added after virus entry, strongly suggests a role of ErbB2 in post entry steps such as viral gene regulation. Here, virus internalization and trafficking has already been completed and freshly added inhibitor might account for the even stronger effect. Indeed, targeting ErbB2 reduced the phosphorylation of this receptor and its downstream signaling pathways, effectively inhibiting LCR activity and eventually infection. This is in line with the previously shown involvement of ErbB2 in Akt and ERK activation, and consequently transcriptional modulation of various genes (102). Moreover, recent investigations revealed that the activation of these pathways via EGFR plays a dominant role in promoting HPV oncogene expression, namely E6 and E7 (103). Our findings also show that ErbB2 plays a major role in mediating the activation of HPV LCRs through classical signal transduction pathways as the observed effect on LCR activity was independent of ErbB2’s nuclear translocation, an alternative way of ErbB2 to modulate gene regulation (39, 40, 43, 44, 62). In addition, we demonstrate that the ErbB2-specific LCR regulation was observed for different HPV types, irrespective of their risk classification, suggesting a conserved mechanism.

Tucatinib and CP-724714 are found to be suitable pharmacological agents for blocking ErbB2-dependent signal transduction in keratinocytes. The observation that higher concentrations of tucatinib might yield stronger reduction in infection warrants further exploration. On the one hand, the current study focused on a specific concentration to avoid non-ErbB2-mediated effects. On the other hand, it is likely that EGFR and ErbB2 receptors act synergistically to activate signaling pathways necessary for efficient HPV infection. Here, the importance of ErbB2 might lie in prolonging and enhancing down-stream signaling when present in the heterodimer with EGFR, whereas EGFR homodimers trigger weak signaling (104). Moreover, the EGFR/ErbB2 dimer is involved in steps that are crucial for cancer progression, such as cell proliferation, migration and invasiveness (45, 48, 104). A slight decrease in EGFR phosphorylation upon ErbB2 depletion or inhibition suggests that EGFR phosphorylation relies to some extent on the presence of ErbB2. Thus, it is plausible that the observed effects on LCR activity may be linked to a decrease in downstream signaling mediated by ErbB2-EGFR heterodimers. Therefore, we speculate that ErbB2 forms a heterodimer with EGFR during virus entry platform formation for inducing prolonged and enhanced Akt and ERK signaling which facilitates the establishment of HPV infection and early gene expression.

Investigations on cervical cancer cell lines CaSki and HeLa containing integrated HPV16 and HPV18 viral genomes, respectively, confirmed ErbB’s role in Akt and ERK signaling and its importance for viral oncogene expression and promoter activity. In cervical cancer, especially in ErbB2-positive cancers, inhibition of ErbB2 activation might lead to the downregulation of the viral early genes including E6 and E7. The HPV oncoproteins are also potent immune modulators of cellular key proteins such as p53 (105–107). Inhibition of the early promoter and viral gene expression in turn leads to the stabilization or increase in e.g., p53 (108), which then causes the induction of genes that can promote intracellular immunity (12, 106, 109). Several p53 target genes are involved in driving IFN production and signaling, including TLR3, IRF5, ISG15 and IRF9 (109). Thus, suppressing the HPV early promoter may provide a positive effect on the intracellular immunity by regulation of viral protein expression. This hypothesis remains to be investigated.

In conclusion, this study uncovered ErbB2 as a host cell factor in HPV infections and its targetable function on HPV promoter activity. More importantly, the FDA-approved ErbB2 inhibitor tucatinib emerges as a promising tool for therapeutic intervention in HPV-associated pathologies by potentially suppressing viral oncogene expression. Future studies and clinical trials involving tucatinib or tucatinib/trastuzumab combinations are eagerly awaited to validate these findings and explore their therapeutic potential further.

## Data availability statement

The original contributions presented in the study are included in the article/**Supplementary Material**. Further inquiries can be directed to the corresponding author.

## Ethics statement

Ethical approval was not required for the studies on humans in accordance with the local legislation and institutional requirements because only commercially available established cell lines were used. Ethical approval was not required for the studies on animals in accordance with the local legislation and institutional requirements because only commercially available established cell lines were used.

## Author contributions

SM: Data curation, Project administration, Validation, Writing – original draft, Writing – review & editing, Investigation, Visualization. MS: Investigation, Writing – review & editing, Conceptualization. AM: Formal Analysis, Investigation, Writing – original draft, Validation, Visualization. A-LF: Investigation, Writing – original draft. KF: Writing – original draft, Investigation. TD: Investigation, Writing – original draft. JS: Visualization, Writing – review & editing. ST: Investigation, Writing – original draft, Data curation, Formal Analysis, Resources. TL: Writing – original draft, Conceptualization, Data curation, Funding acquisition, Project administration, Resources, Supervision, Writing – review & editing. LF: Conceptualization, Writing – review & editing, Data curation, Funding acquisition, Project administration, Resources, Supervision, Validation, Writing – original draft.
