# Corrigendum: ErbB2/HER2 receptor tyrosine kinase regulates human papillomavirus promoter activity

**DOI:** 10.3389/fimmu.2024.1385729

**Published:** 2024-02-29

**Authors:** Snježana Mikuličić, Merha Shamun, Annika Massenberg, Anna-Lena Franke, Kirsten Freitag, Tatjana Döring, Johannes Strunk, Stefan Tenzer, Thorsten Lang, Luise Florin

**Affiliations:** ^1^Institute for Virology, University Medical Center of the Johannes Gutenberg-University Mainz, Mainz, Germany; ^2^University of Bonn, Faculty of Mathematics and Natural Sciences, Life & Medical Sciences (LIMES) Institute, Bonn, Rheinland-Pfalz, Germany; ^3^Institute for Immunology, University Medical Center of the Johannes Gutenberg-University Mainz, Mainz, Rheinland-Pfalz, Germany; ^4^Helmholtz Institute for Translational Oncology (HI-TRON) Mainz, Mainz, Rheinland-Pfalz, Germany

**Keywords:** human papillomavirus, HPV16, promoter activity, ErbB2, HER2/neu, tyrosine kinase inhibitor, tucatinib, E6 E7 oncogene expression

In the published article, there was an error in **Figure 7** as published. The x-axis labels in **Figure 7B** and **Figure 7D** were listed as ‘contr.’ and ‘T’, respectively. However, the correct x-axis labels should be ‘contr.’ and ‘CP’, as these two graphs display data from CP-724714-treated cells (CP) and not from Tucatinib-treated cells (T). The corrected **Figure 7** and its caption appear below.

**Figure 7:**
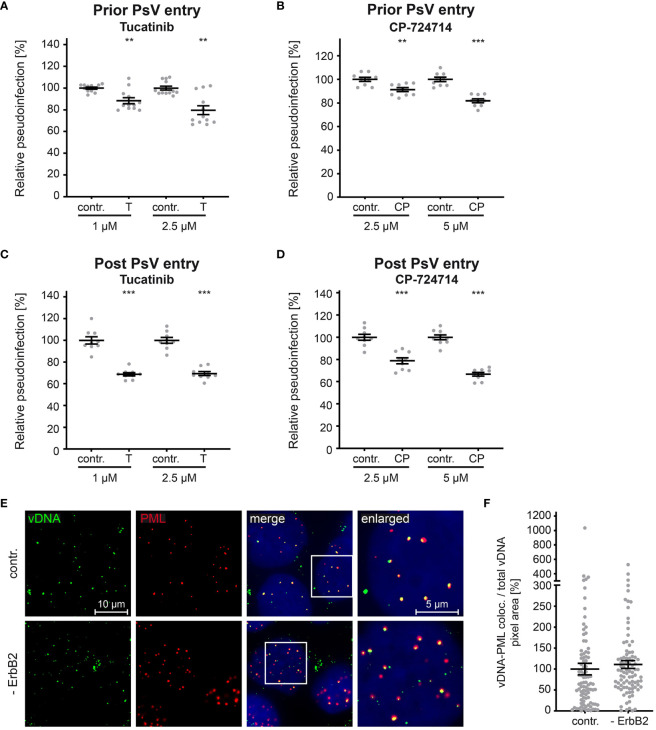
ErbB2 affects post entry steps of HPV PsVs infection. **(A, B)** HaCaT cells were treated with solvent control (contr.) or the indicated concentration (in μM) of tucatinib (T) or CP-724714 (CP) for 1 h and subsequently exposed to HPV16 PsVs. The luciferase and the LDH activities were assessed 24 h later. **(A)** Effect of tucatinib. The statistical difference between the two groups (n = 12) was analyzed with the Mann-Whitney test (p = 0.0042 for contr. 1 vs. T 1 and contr. 2.5 and T 2.5). **(B)** Effect of CP-724714. The statistical difference between the two groups (n = 9) was analyzed with the Welch’s t test (p = 0.0025 for contr. 2.5 and CP 2.5; p < 0.0001 for contr. 5 and CP 5). **(C, D)** HaCaTs were infected with HPV16 PsVs and 24 h later treated with control (contr.) or the indicated concentration of tucatinib or CP-724714 for another 5 h to enable mRNA and protein turn-over after ErbB2 signaling inhibition. **(C)** Effect of tucatinib. The statistical difference between the two groups (n = 9) was analyzed with the Welch’s t test (p < 0.0001 for contr. 1 vs. T 1 and contr. 2.5 vs. T 2.5). **(D)** Effect of CP-724714. The statistical difference between the two groups of interest (n = 9) was analyzed with the Welch’s t test (p < 0.0001 for contr. 2.5 vs. CP 2.5 and contr. 5 vs. CP 5). Relative pseudoinfection was normalized to LDH. Data are given as means ± SEM, and the mean for contr.-treated cells set to 100%. **(E)** Representative images of HaCaT cells transfected either with control (contr.) or two ErbB2 targeting siRNA, siRNA#1 and #3 (- ErbB2) and after 48 h incubated with HPV16 PsVs for 24 h. EdU-PsVs (green) were visualized by click-labeling of the plasmid DNA, whereas PML (red) with anti-PML antibody. Image acquisition was performed using a Zeiss Axiovert 200 M microscope fitted with a Plan-Apochromat 100Å~/1.4 Oil objective (Carl Zeiss, Jena, Germany). Quantification of colocalization was performed by analysis of at least 20 pictures per group using Colocalization Software 4.7 (Carl Zeiss). **(F)** Relative colocalization of vDNA and PML. vDNA pixels colocalizing with PML pixels are given as means ± SEM, and the mean for control siRNA-treated cells (contr.) was set to 100%. p ≤ 0.01 **, p ≤ 0.001 ***.

In the published article, there was an error. The CD151-specific antibody used for the WB was not described correctly. A correction has been made to Section 2 Materials and methods, Sub-section Antibodies, inhibitors, and plasmids, Paragraph 1. This sentence previously stated: ‘A rabbit pAb raised against CD151 (ab185684) used for STED experiments was purchased from (Abcam, Amsterdam, Netherlands) and CD151-specific mouse mAb antibody (11G5a) for WB from Bio-Rad (Munich, Germany).’ The corrected sentence appears below:

‘A rabbit pAb raised against CD151 (ab185684) used for STED experiments was purchased from Abcam (Amsterdam, Netherlands) and a rabbit anti-CD151 serum generated against the recombinant large extracellular loop of CD151 (rCD151) used for WB after non-reducing SDS-PAGE was a kind gift from Fedor Berditchevski (University of Birmingham, United Kingdom).’

The authors apologize for these errors and state that this does not change the scientific conclusions of the article in any way. The original article has been updated.

