# Supplementary material for: ErbB2/HER2 receptor tyrosine kinase regulates human papillomavirus promoter activity
Source: Front Immunol. 2024 Feb 2;15:1335302. doi: 10.3389/fimmu.2024.1335302 (PMC10869470; doi:10.3389/fimmu.2024.1335302)
Supplement: Supplementary file 1 [file DataSheet_1.docx]

Supplementary Material

# Supplementary Figures and Tables

**Supplementary Table 1.** Quantitative mass spectrometry (MS) analysis of HaCaT and NHEK endosomal fractions. MS detected enrichment of cellular proteins in endosomal fractions after cells’ exposure to HPV16 PsVs. The endosomal fractions from NHEK and HaCaT cells and different time points (untreated, 4h, 7h post PsVs addition) were analyzed. Parts per million (ppm) values are calculated at the protein levels. The amount of each protein is determined using the 3 “best ionizing” peptides, which are then compared to the total protein in the sample. Each preparation was analyzed in five technical replicates. The proteins were reproducibly detected in at least three out of five technical replicates and are shown as mean values. The proteins are sorted according to the fold change of the mean values comparing the 7 h post PsV addition time point and untreated samples. Non-detects were assumed as ppm = 1. Cellular proteins which were detected as 2-fold enriched or higher at 7h post PsVs addition compared to the untreated control are displayed on a blue background. Results for ErbB2, LAMP1 and EGFR are highlighted with a frame.
